# Supplementary material for: Catalytically inactive long prokaryotic Argonaute systems employ distinct effectors to confer immunity via abortive infection
Source: Nat Commun. 2023 Nov 1;14:6970. doi: 10.1038/s41467-023-42793-3 (PMC10620215; doi:10.1038/s41467-023-42793-3)

Supplemental information for

Catalytically inactive long prokaryotic Argonaute systems employ distinct effectors to confer immunity via abortive infection

Xinmi Song, Sheng Lei, Shunhang Liu, Yanqiu Liu, Pan Fu, Zhifeng Zeng, Ke Yang, Yu Chen, Ming Li, Qunxin She, Wenyuan Han

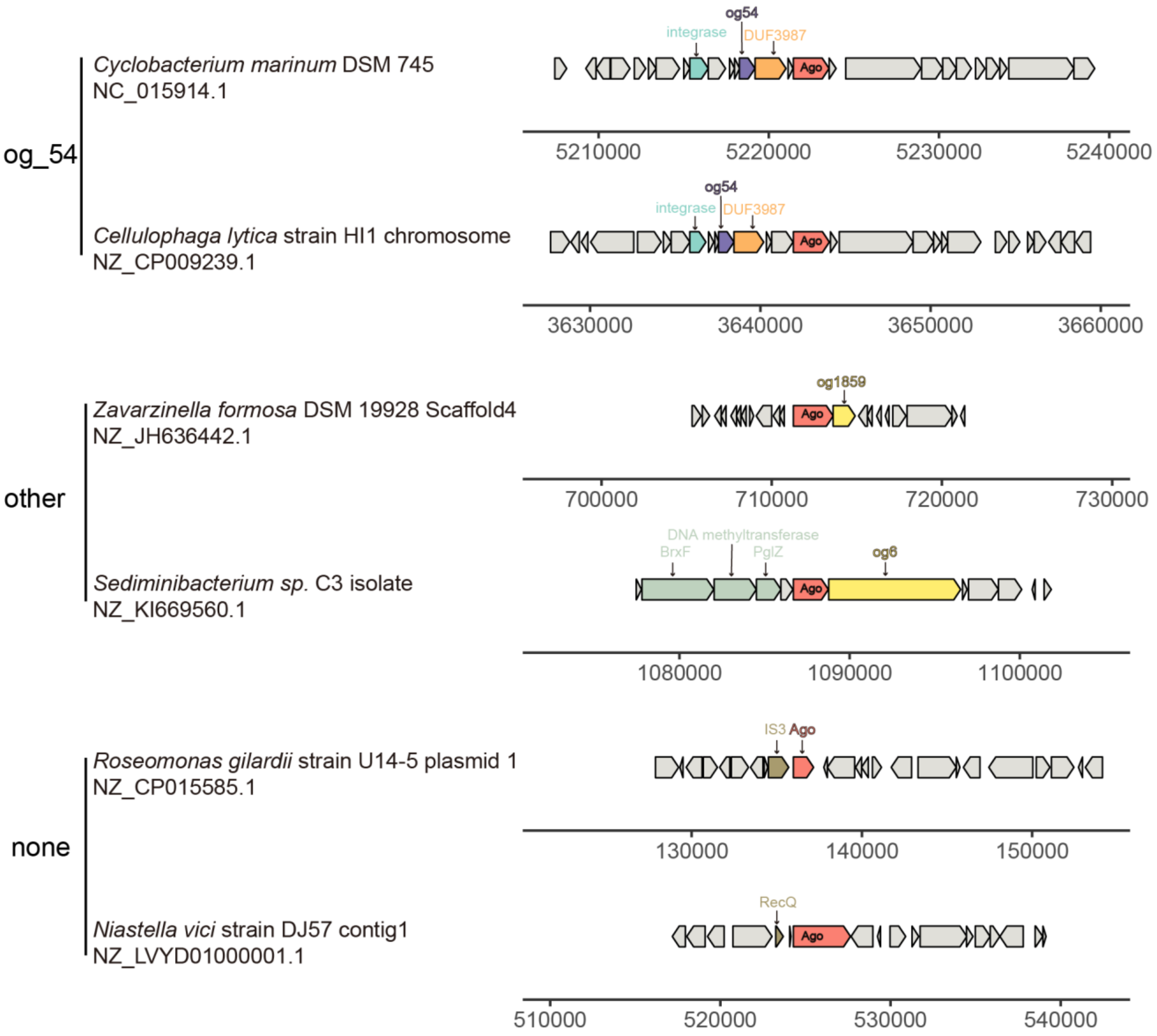

Supplementary Fig. 1. Representative gene clusters of the long-B pAgos that are associated with og_54 and other ogs, and have no associated proteins.

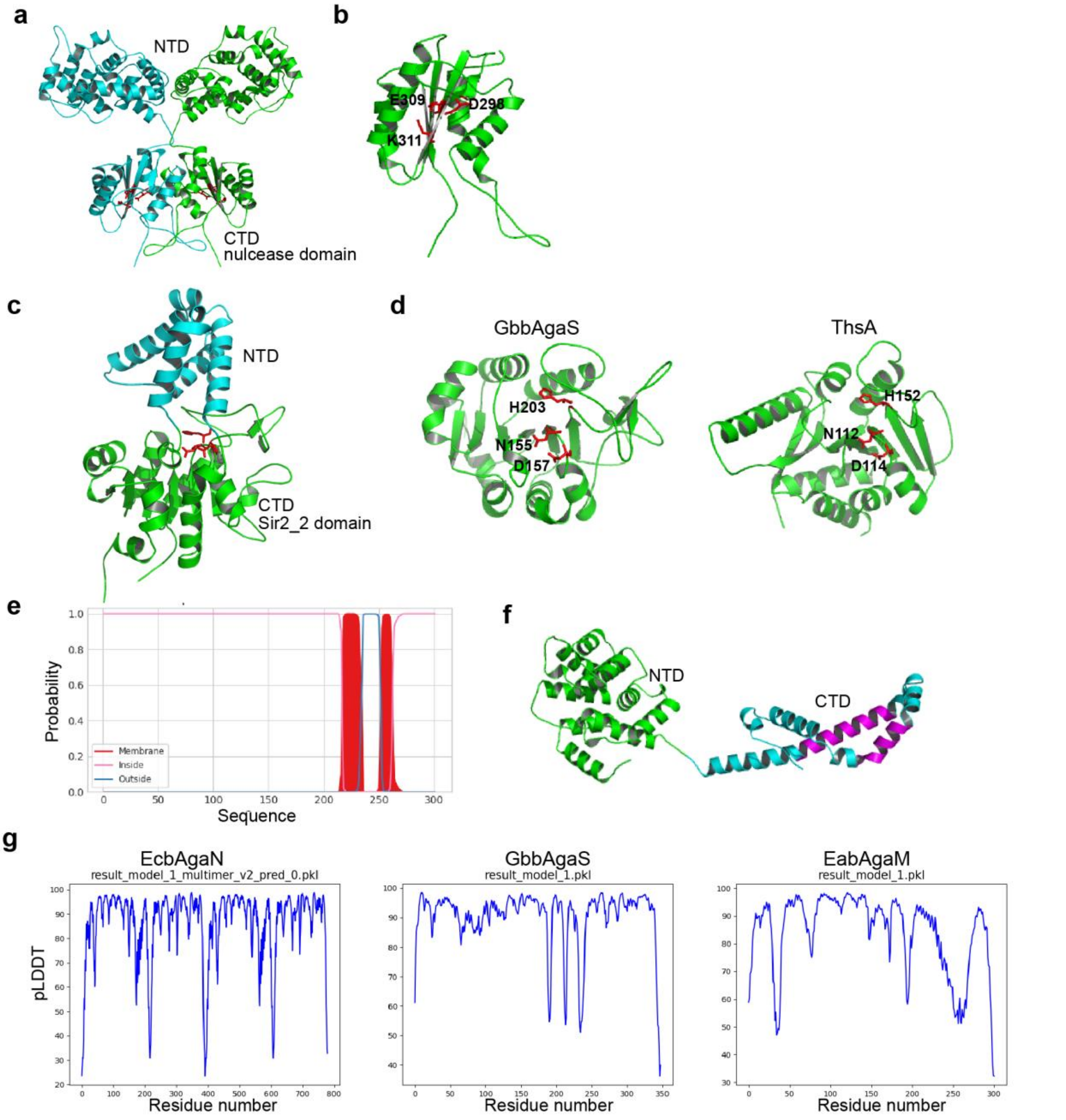

Supplementary Fig. 2. Structural analysis of long-B pAgo-associated proteins

(a) Structural prediction of the EcbAgaN dimer by AlphaFold2. The N-terminal domain (NTD) and the C-terminal domain (CTD, the nuclease domain) are indicated

(b) A close-up view of the nuclease domain of EcbAgaN. The predicted catalytic sites are indicated.

(c) Structural prediction of GbbAgaS by AlphaFold2. The CTD is the Sir2_2 domain.

(d) Structural comparison of the Sir2_2 domain of GbbAgaS and the Sir2 domain of ThsA (PDB: 6LHX). The catalytic sites are shown.

(e) Transmembrane prediction of EabAgaM by DeepTMHMM.

(f) Structural prediction of EabAgaM by AlphaFold2. The predicted transmembrane region is shown in magenta.

(g) The pLDDT values of the AlphaFold2 models of EcbAgaN, GbbAgaS and EabAgaM.

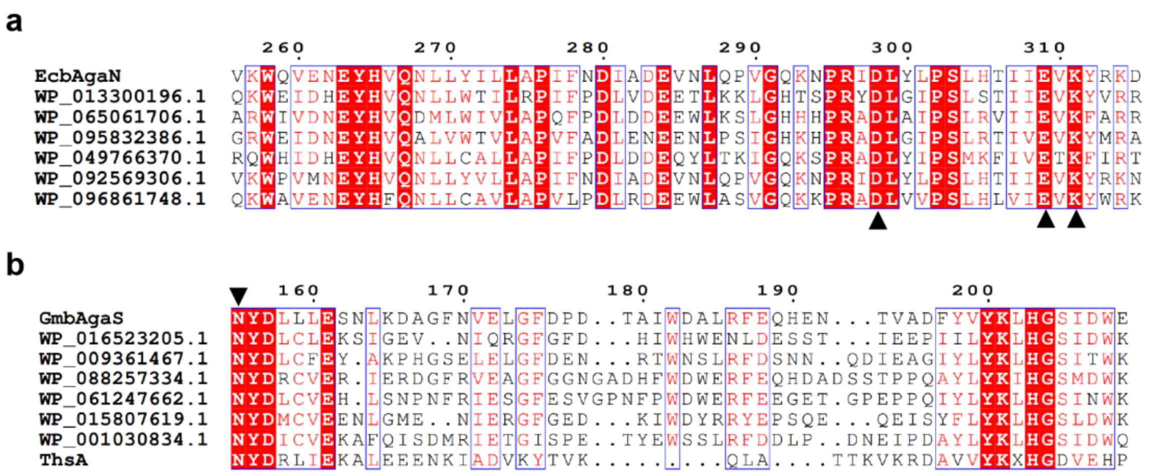

Supplementary Fig. 3.

(a) Sequence alignment of EcbAgaN and other representative og_15 members.

(b) Sequence alignment of GbbAgaS and other representative og_44 members, as well as ThsA.

Only the conserved region containing the catalytic sites is shown. The arrows indicate the residues for mutagenesis.

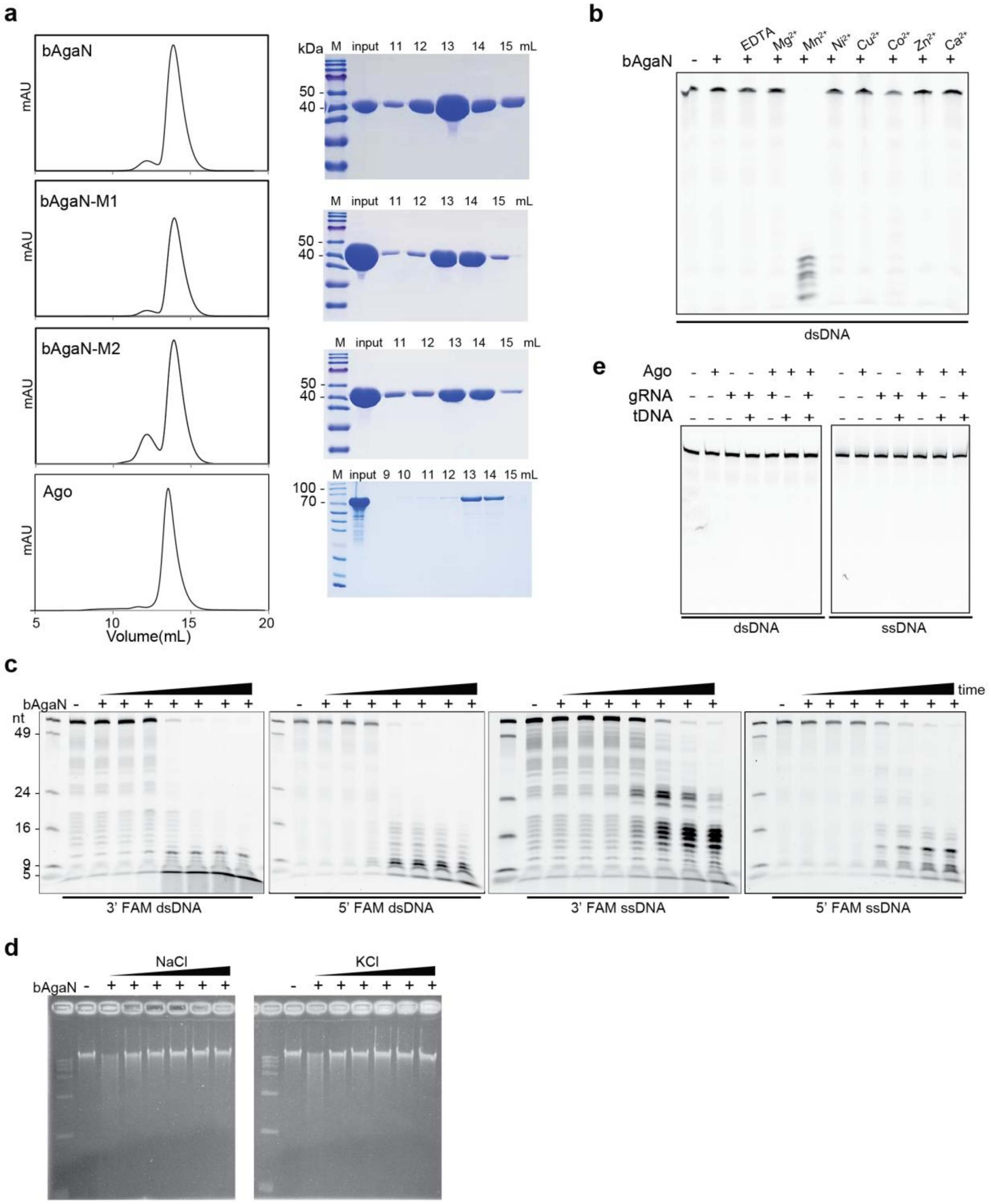

Supplementary Fig.4 Purification and *in vitro* characterization of EcbAgaN and EcAgo.

(a) Gel filtration profiles of purified EcbAgaN, its mutants and EcAgo (left panels), and SDS-PAGE analysis of the gel filtration samples (right panels). Input: the samples that were loaded onto the gel filtration column. M: protein marker.

(b) Metal-dependency of EcbAgaN. FAM-labeled dsDNA was incubated with EcbAgaN in the presence of EDTA or indicated metal ions, and then analyzed by denaturing polyacrylamide gel electrophoresis.

(c) Time course of ssDNA and dsDNA degradation of EcbAgaN. Time points: 0, 1, 2, 5, 10, 20, 40 min.

(d) The DNase activity of EcbAgaN is inhibited by NaCl and KCl. Genomic DNA was degraded by EcbAgaN in the presence of a gradient of NaCl and KCl concentrations respectively. NaCl concentrations: 25, 75, 125, 175, 225, 325 mM; KCl concentrations: 0, 50, 100, 150, 200, 300 mM.

(e) EcAgo does not cleave target ssDNA or dsDNA. FAM-labeled target ssDNA or dsDNA was incubated with EcAgo. Guide RNA and/or non-labeled target ssDNA were also supplemented in aliquots of the reaction. Then, the samples were analyzed by denaturing polyacrylamide gel electrophoresis.

Source data are provided as a Source Data file.

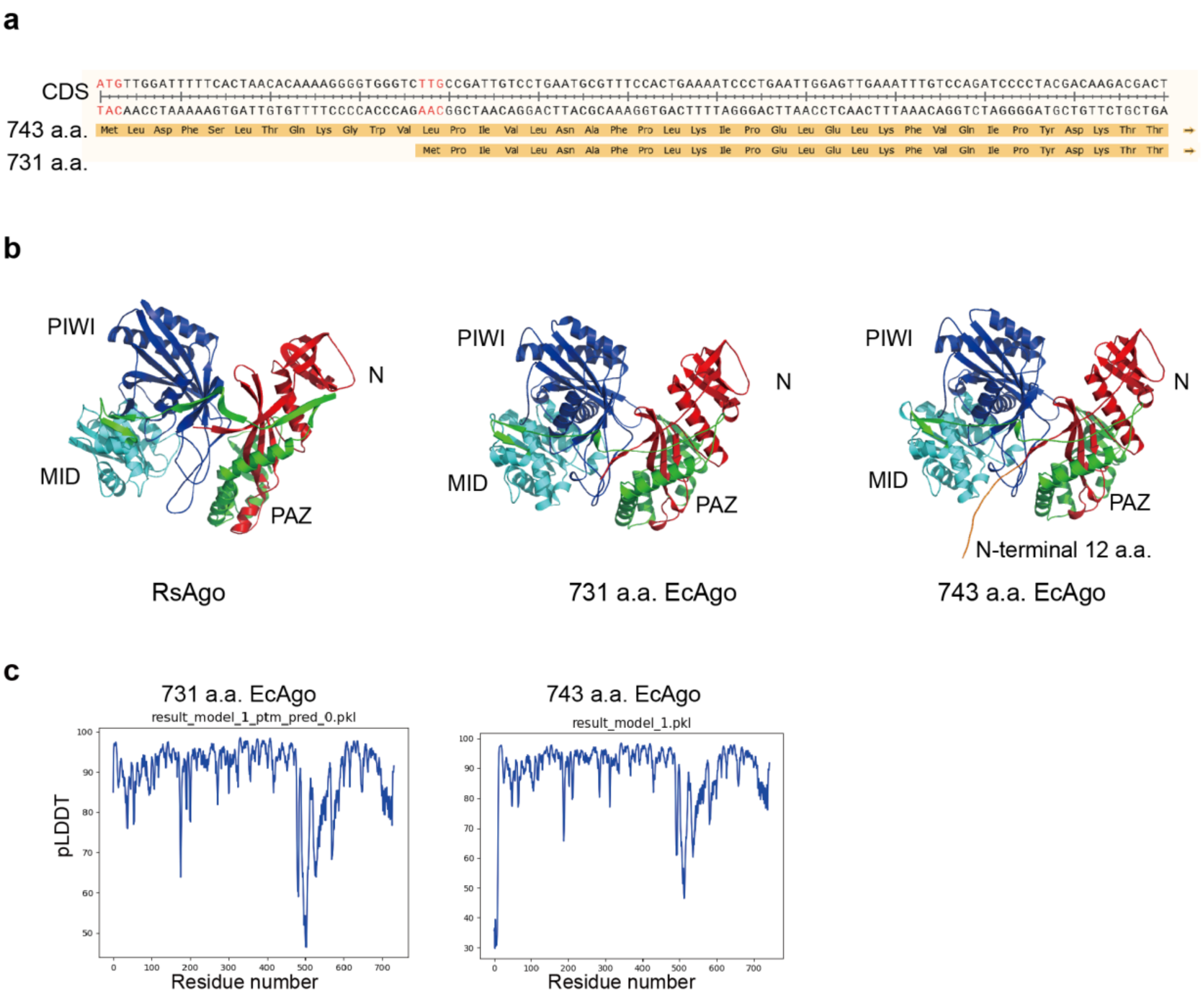

Supplementary Fig. 5 Structural analysis of EcAgo.

(a) *EcAgo* has two predicted starting codons, and the encoded proteins have 743 amino acids (a.a.) and 731 a.a. respectively. The coding sequence (CDS) and a.a. sequences of the N-terminal region are shown.

(b) Structural comparison of the predicted structures of EcAgo and RsAgo (PDB: 5AWH). Different domains and the N-terminal 12 a.a. of the 743 version EcAgo are shown in different colors.

(c) The pLDDT values of the AlphaFold2 models of the two version of EcAgo.

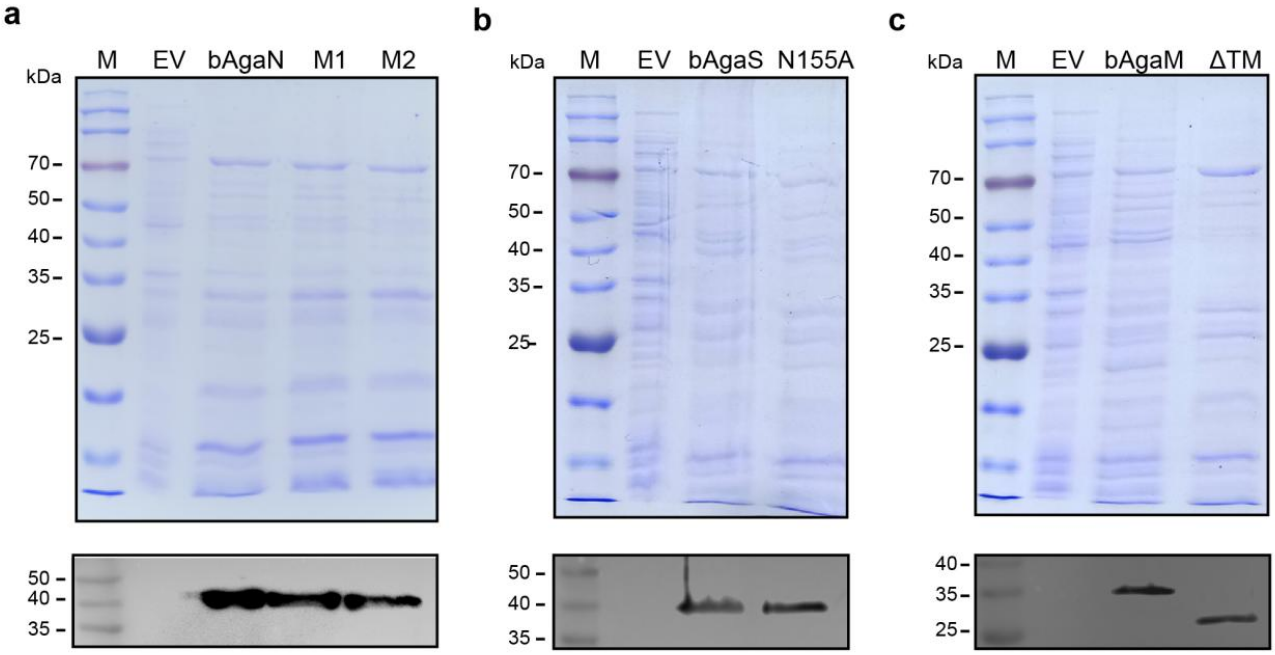

Supplementary Fig. 6 Western blot analysis of wild type and mutated EcbAgaN (a), GbbAgaS (b) and EabAgaM (c), respectively.

Cell extracts from the cells containing empty vector (EV) or expressing the indicated proteins were prepared and analyzed by SDS-PAGE (upper panel) and western blot using anti-His-tag antibody (bottom panel).

Source data are provided as a Source Data file.

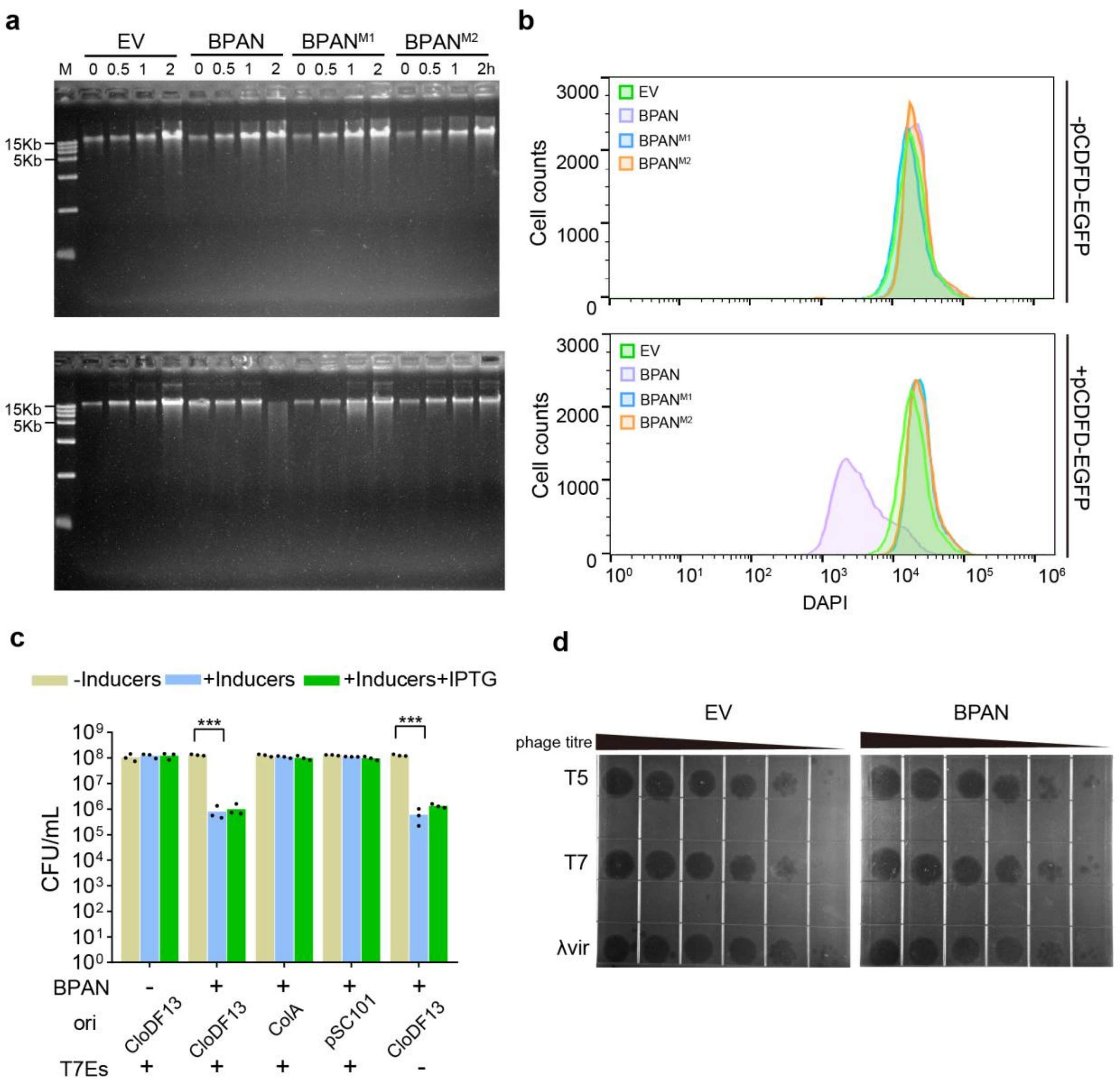

Supplementary Fig. 7 *In vivo* characterization of the EcBPAN system.

(a) pCDF-EGFP activates the BAC (bacterial artificial chromosom)-expressed EcBPAN system to mediate genomic DNA degradation. The cells expressing wild type and mutated EcBPAN systems (M1 and M2) in the absence (upper panel) or presence (bottom panel) of pCDF-EGFP were grown in LB medium supplemented with L-arabinose. At indicated time points, genomic DNA was extracted and analyzed by agarose gel electrophoresis.

(b) Flow cytometry analysis of DNA content distributions in the cells after the wild type and mutated EcBPAN systems were induced by L-arabinose for 2 h in the absence (upper panel) or presence (bottom panel) of pCDF-EGFP.

(c) The pBAD24-expressed EcBPAN system is activated by the CloDF13 origin. The cells expressing EcBPAN system using pBAD24 were transformed with pCDF-EGFP and its variants, where the CloDF13 origin was substituted with the indicated origins or the T7 expression cassettes (T7Es) were removed. Then, the cells were plated onto the plates with or without inducers, or with inducers and IPTG. Inducer: L-arabinose and aTc. Data are presented as mean values with individual data points overlaid (n = 3 biological replicates). The p

values were calculated using one-sided Student’s T-Test. ***: $p < 0.001$; p=0.00004972, 0.0001242 (from left to right).

(d) EcBPAN system does not confer immunity against selected phages. The phages were serially diluted and dropped the bacterial lawns expressing EcBPAN system or the mutated systems.

EV: empty vector.

Source data are provided as a Source Data file.

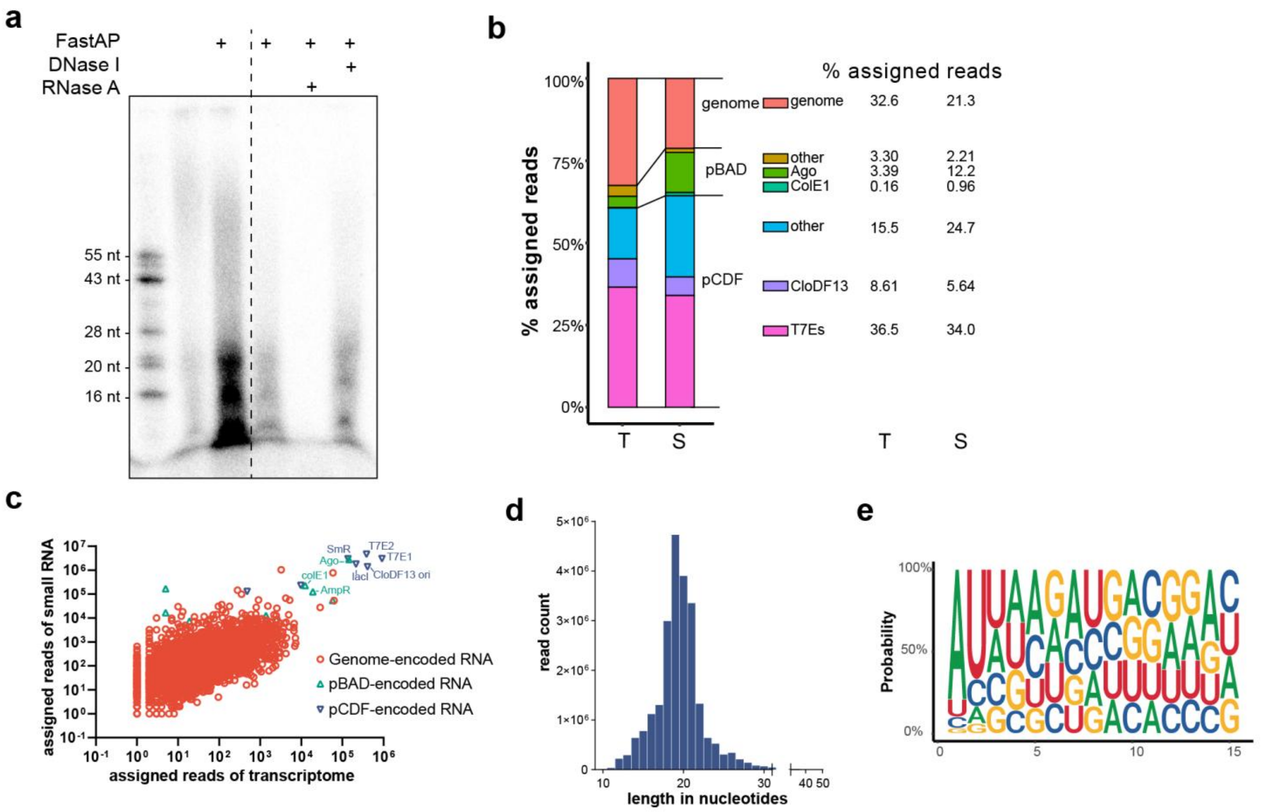

Supplementary Fig. 8 Analysis of the EcAgo-associated small RNAs from the cell containing pBAD24-EcAgo and pCDF-EGFP.

(a) The nucleic acids were treated with FastAP or not, and then labeled with γP^{32}-ATP and analyzed by denaturing polyacrylamide gel electrophoresis. The labeled nucleic acids were also analyzed by DNase I and RNase A treatment.

(b) Percentages of the transcriptome sequences (T) and small RNA sequences (S) that are assigned to genome and specific plasmid elements. The elements are indicated with different colors, with the percentages shown on the right.

(c) Correlation between the transcriptome sequences and small RNA sequences. The Pearson correlation coefficient is $r > 0.77$ with p value $< 10^{-99}$. The p value was calculated with two-sided t-test.

(d) Length distributions of the small RNAs.

(e) Nucleotide bias of the small RNAs.

Source data are provided as a Source Data file.

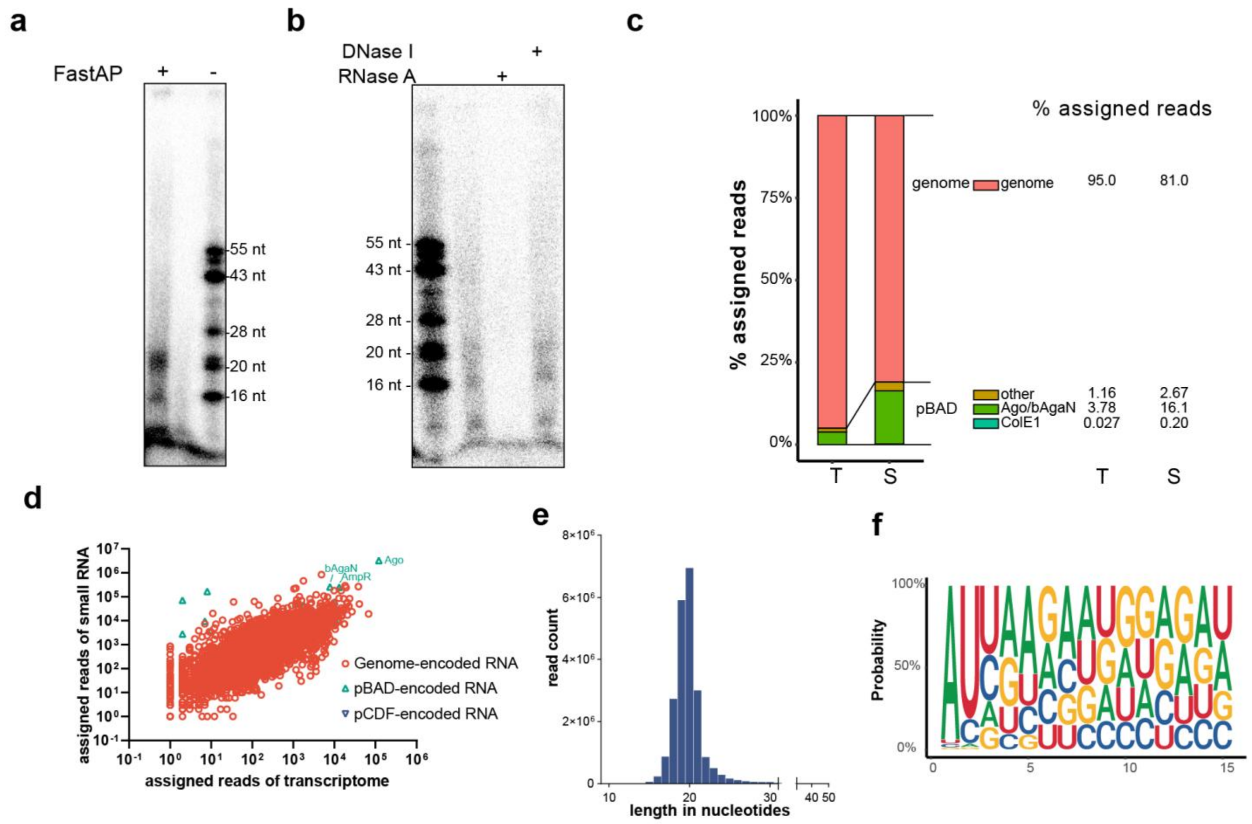

Supplementary Fig. 9 Analysis of the EcAgo-associated small RNAs from the cell containing pBAD24-EcAgo-EcbAgaN.

(a) The nucleic acids were treated with FastAP or not, and then analyzed by γP^{32}-ATP labeling.

(b) Treatment of the labeled nucleic acids with DNase I and RNase A, respectively.

(b) Percentages of the transcriptome sequences (T) and small RNA sequences (S) that are assigned to genome and specific plasmid elements. The elements are indicated with different colors, with the percentages shown on the right.

(d) Correlation between the transcriptome sequences and small RNA sequences. The Pearson correlation coefficient is r > 0.75 with p value < 10^{-99}. The p value was calculated with two-sided t-test.

(e) Length distributions of the small RNAs.

(f) Nucleotide bias of the small RNAs.

Source data are provided as a Source Data file.

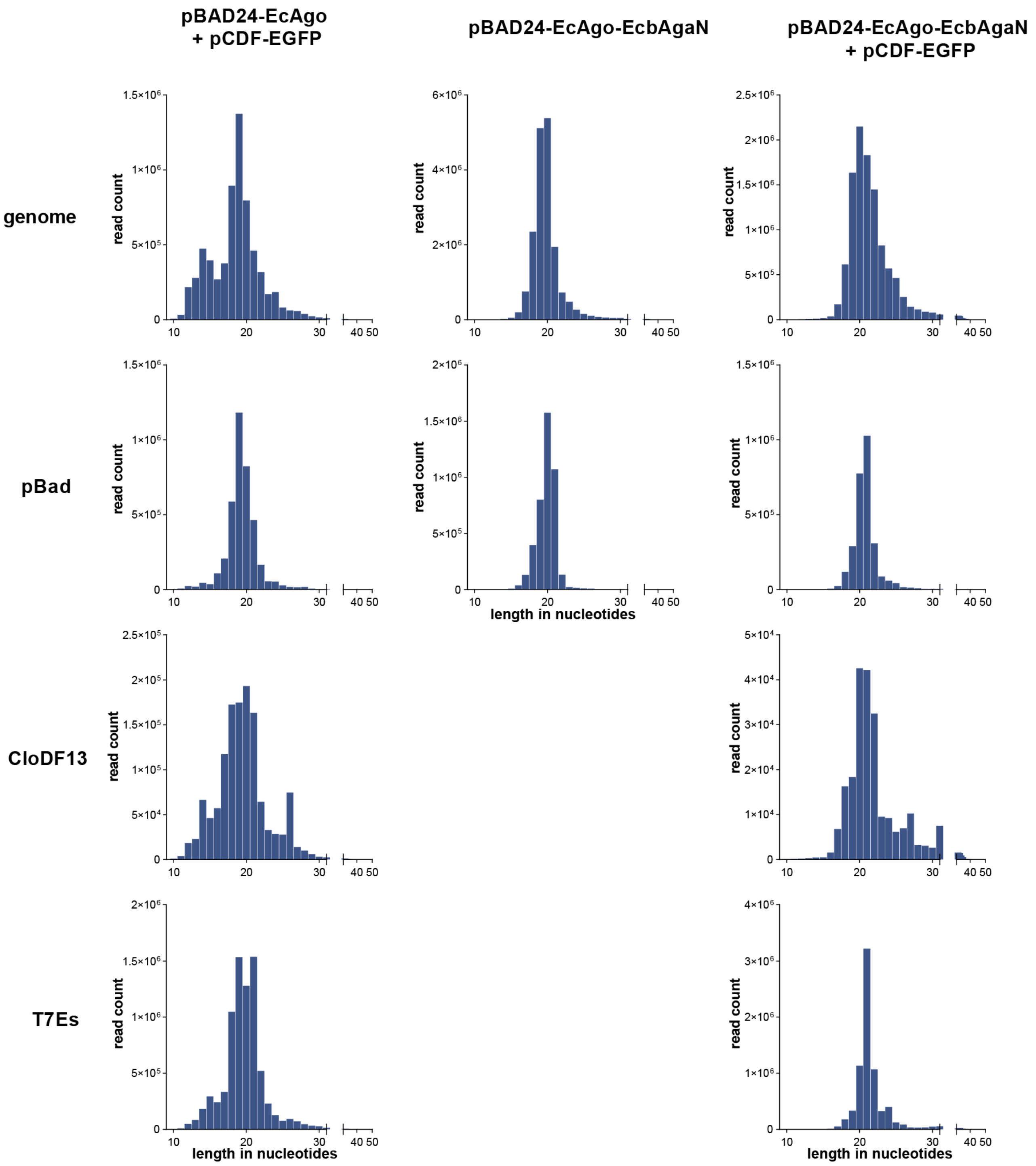

Supplementary Fig. 10 Length distribution of the EcAgo-associated small RNAs assigned to genome, pBAD plasmids, and the CloDF13 origin and the T7 expression cassettes (T7Es) of the pCDF-EGFP plasmid. The small RNAs were from three samples, as indicated by the plasmids carried by the cells. pBAD can be pBad24-EcAgo or pBad24-EcAgo-EcbAgaN, depending on the samples.

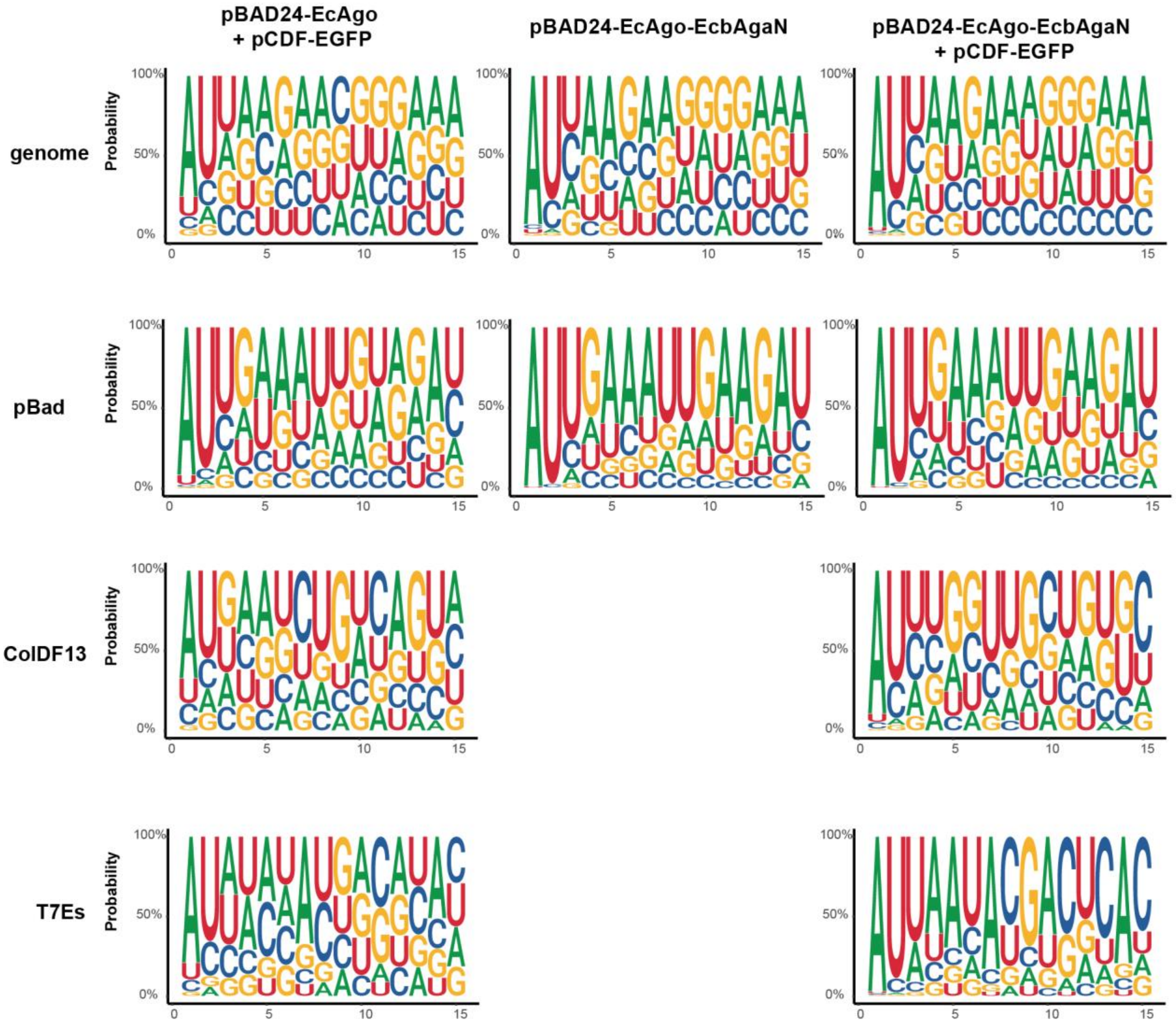

Supplementary Fig. 11 Nucleotide bias of the EcAgo-associated small RNAs assigned to genome, pBAD plasmids, and the CloDF13 origin and the T7 expression cassettes (T7Es) of the pCDF-EGFP plasmid. The small RNAs were from three samples, as indicated by the plasmids carried by the cells. pBAD can be pBad24-EcAgo or pBad24-EcAgo-EcbAgaN, depending on the samples.

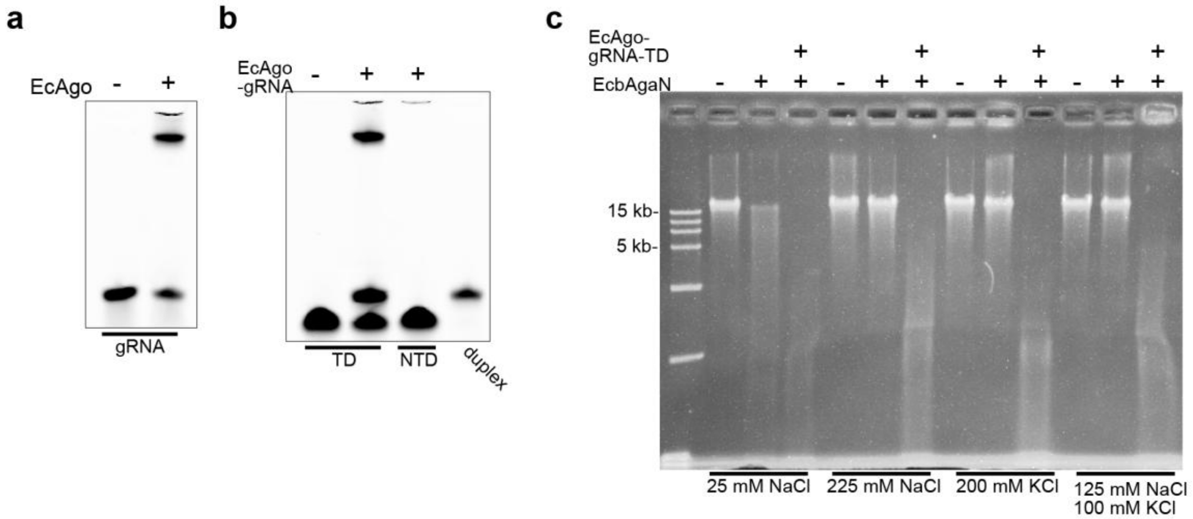

Supplementary Fig. 12

(a) EcAgo binds gRNA in the reaction mixture used in the EcbAgaN activation assay.

(b) EcAgo, gRNA and TD form a ternary complex in the reaction mixture used in the EcbAgaN activation assay.

(c) Effects of NaCl and KCl on the basal activity and the activated activity of EcbAgaN.

Source data are provided as a Source Data file.

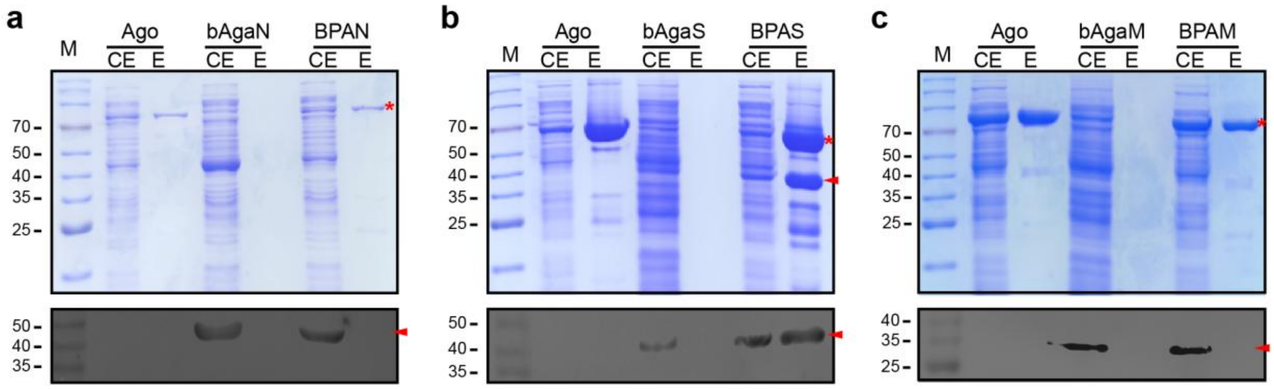

Supplementary Fig. 13 Co-expression and pull-down assay to analyze the interaction between pAgos and their associated proteins. (a-c): EcBPAN, GbBPAS and EaBPAM respectively.

Cell extracts (CE) were prepared from the cells expressing His-tagged Ago, HA-tagged associated protein, or both of them, and then subjected to Ni-NTA affinity chromatography (NAC). Then, CE and the eluates (E) of NAC were analyzed by SDS-PAGE (upper panel) and western blot using anti-HA-tag antibody (bottom panel). pAgos are marked by stars, while the bands of the associated proteins are indicated by arrows.

Source data are provided as a Source Data file.

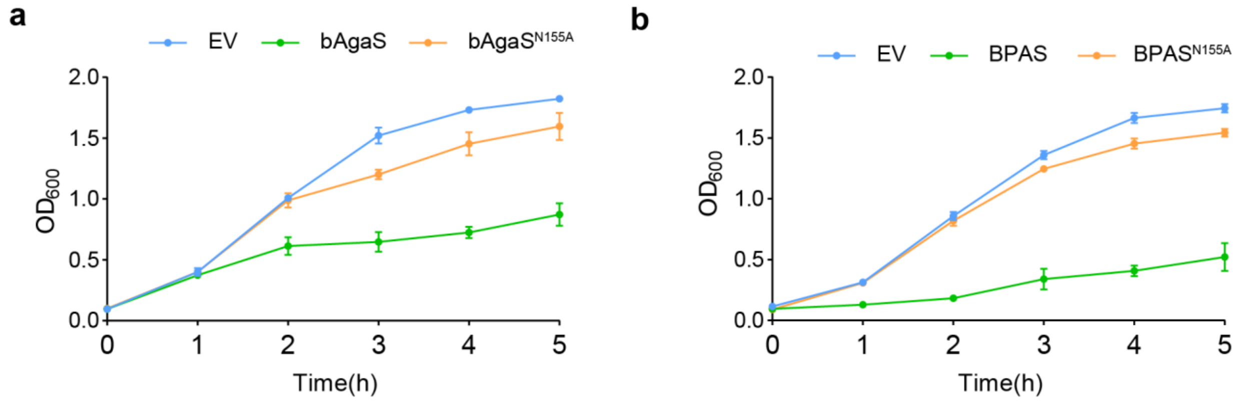

Supplementary Fig. 14

(a) Growth curves of the cultures carrying EV, wild type and mutant GbbAgaS proteins.

(b) Growth curves of the cultures carrying EV, wild type and mutant GbBPAS systems in the presence of pCDF-EGFP.

EV: empty vector.

Source data are provided as a Source Data file.

Supplementary Data 1 Proteins analyzed in the study. The names, accession numbers, source strains, and sequences are listed.

Supplementary Data 2 Plasmids used in the study.

Supplementary Data 3 Primers used in the study.

Supplementary Data 4 Oligonucleotides used in the study.

Supplementary Data 5 Alignments of the sequenced RNA reads from transcriptome and EcAgo-associated small RNAs. The RNAs were from three different samples, as indicated by the plasmids carried by the cells.

Source Data Supplementary Fig. 4b

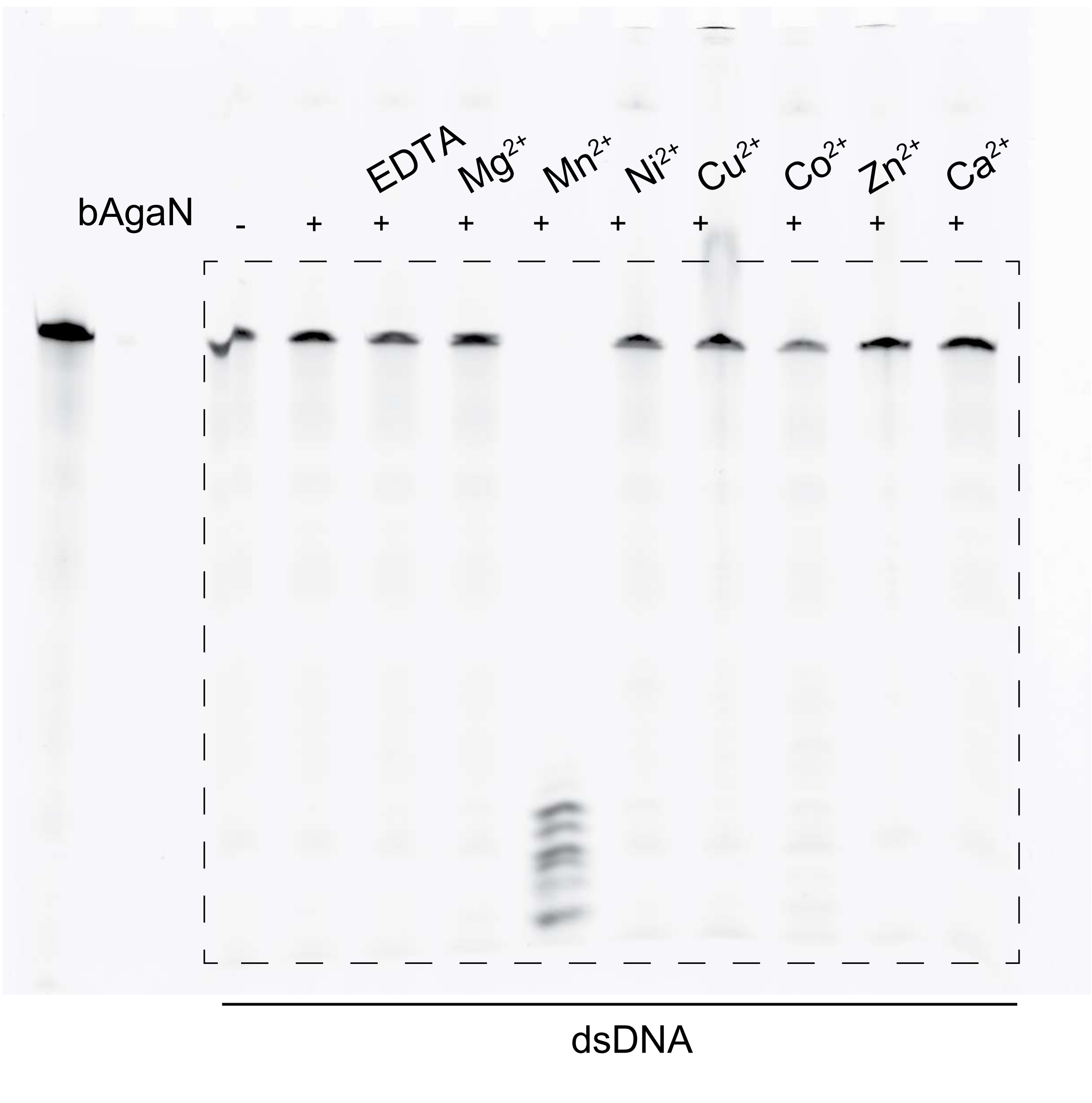

Source Data Supplementary Fig. 4c

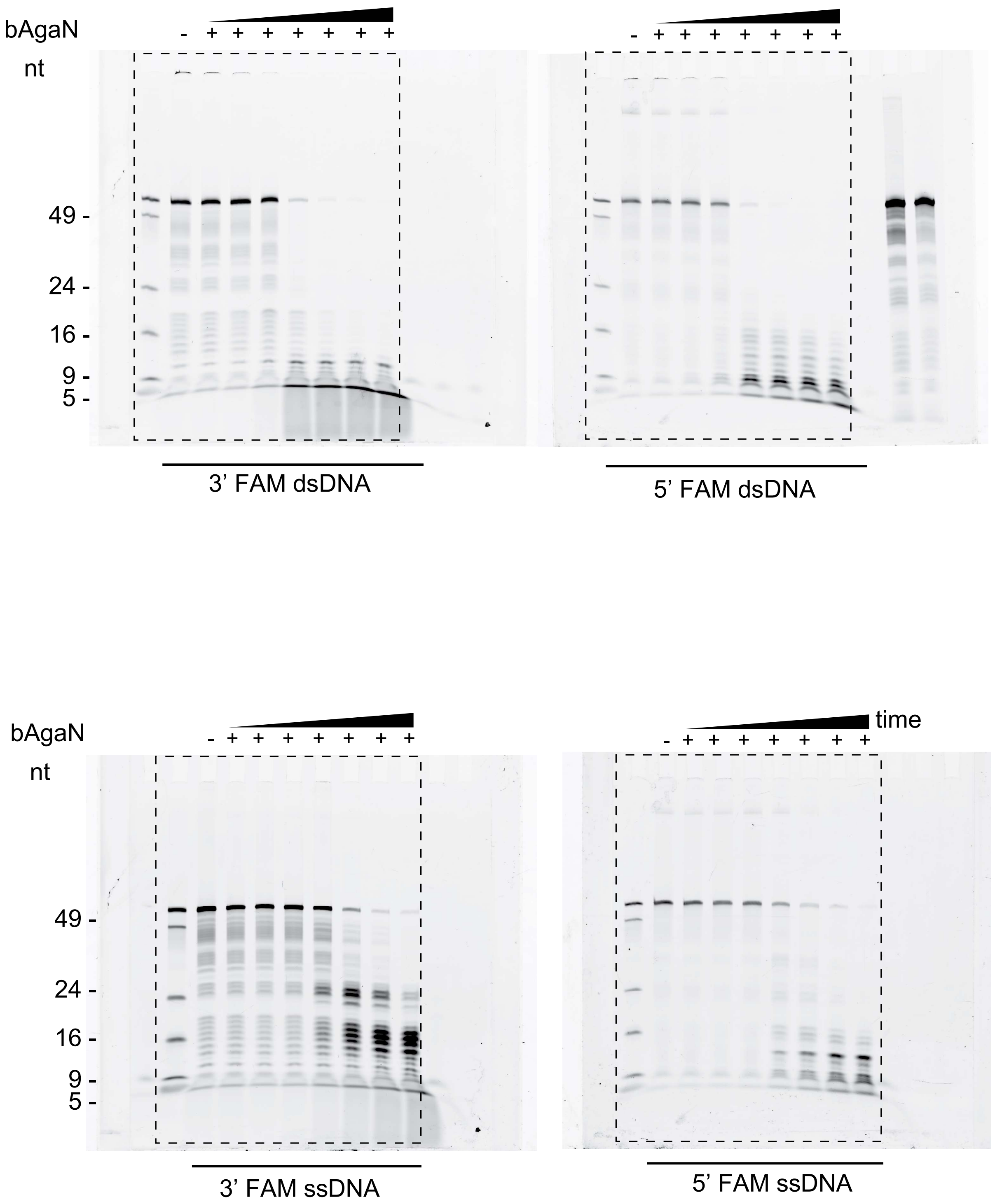

Source Data Supplementary Fig. 4d

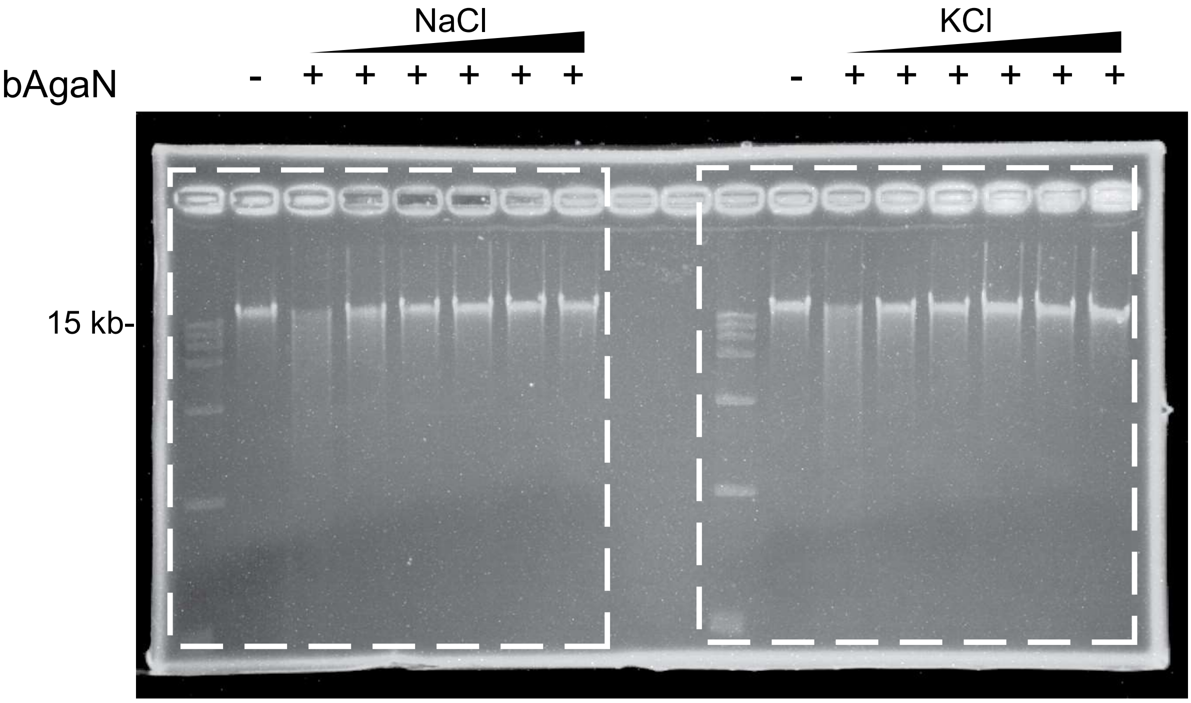

Source Data Supplementary Fig. 4e

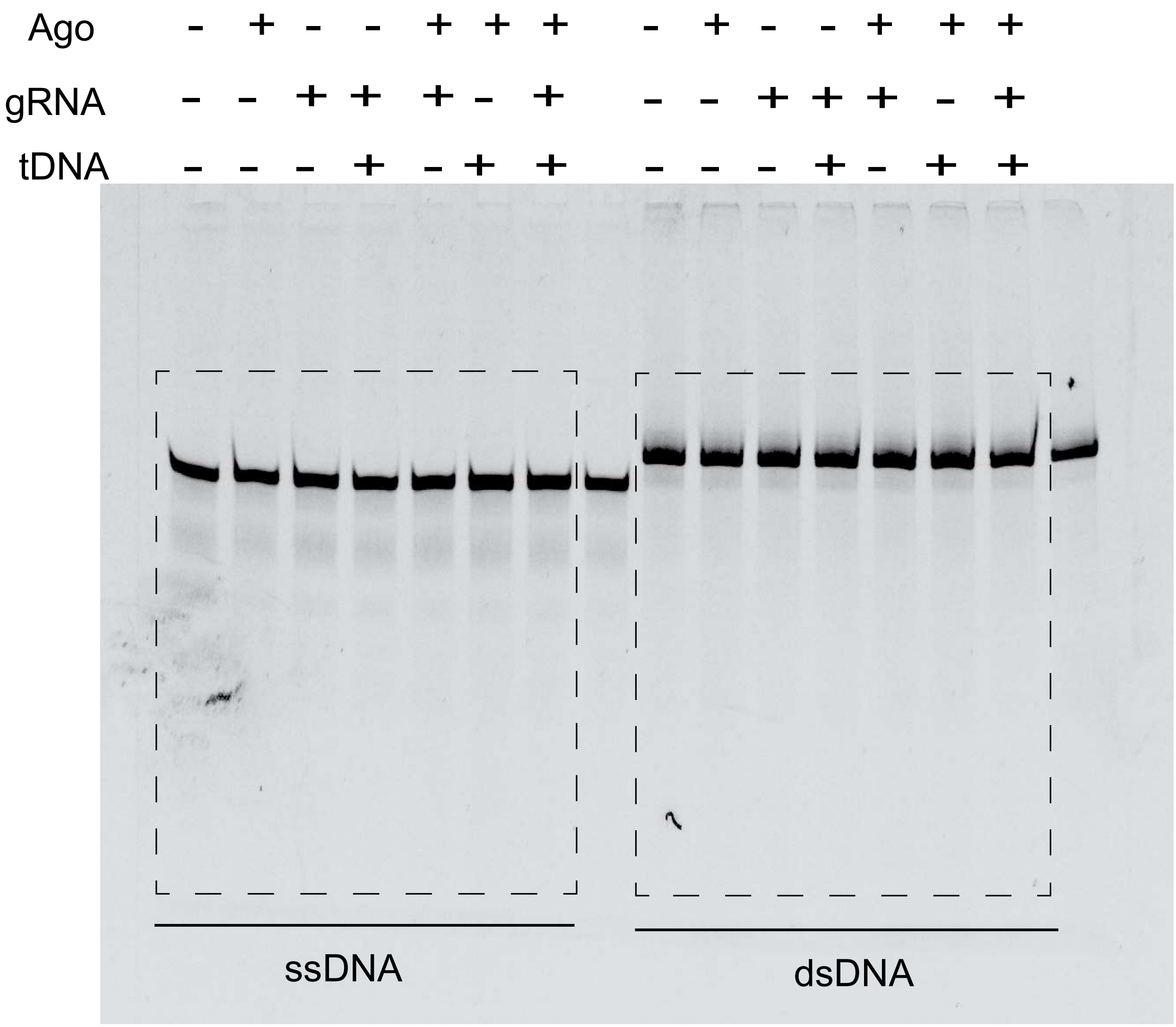

Source Data Supplementary Fig. 6

M EV bAgaN M1 M2

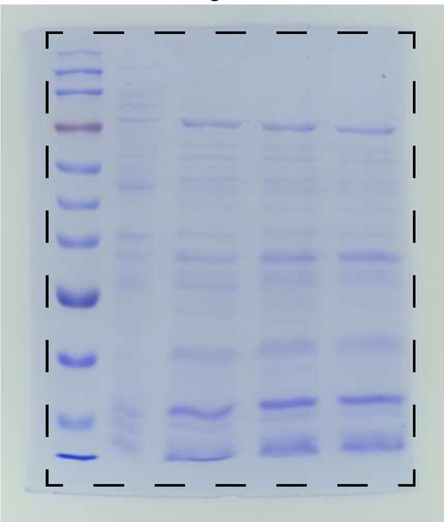

M EV bAgaM ΔTM M EV bAgaS N155A

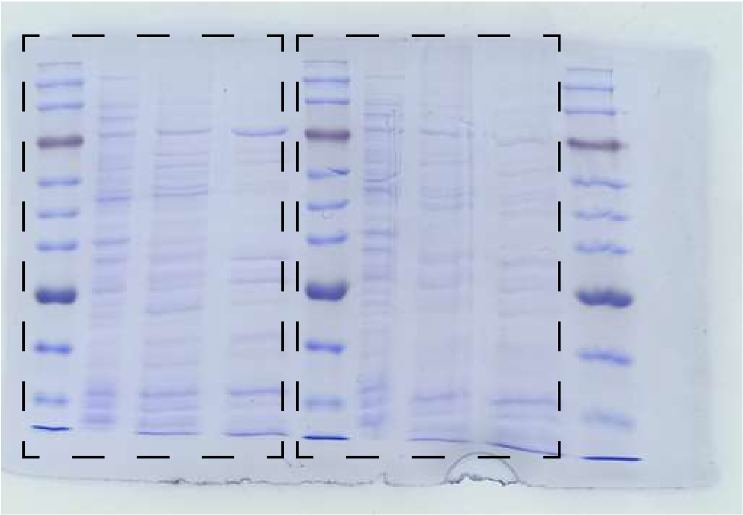

M EV bAgaN M1 M2

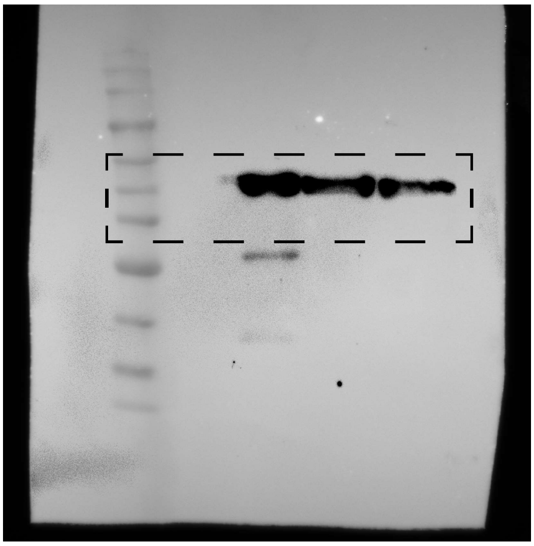

M EV bAgaM ΔTM

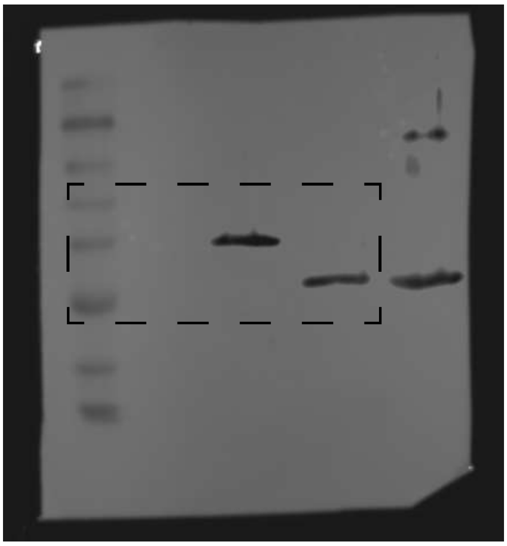

M EV bAgaS N155A

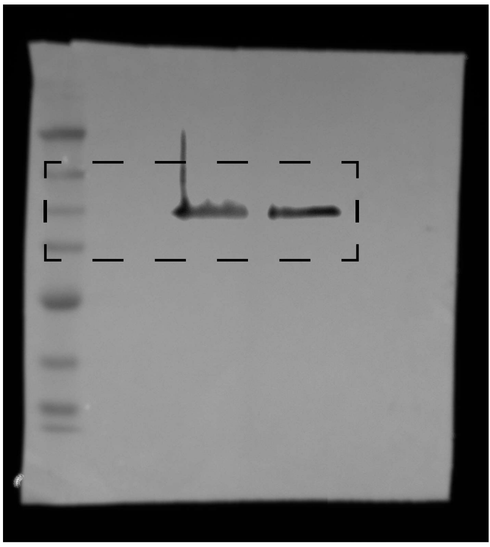

Source Data Supplementary Fig. 7a

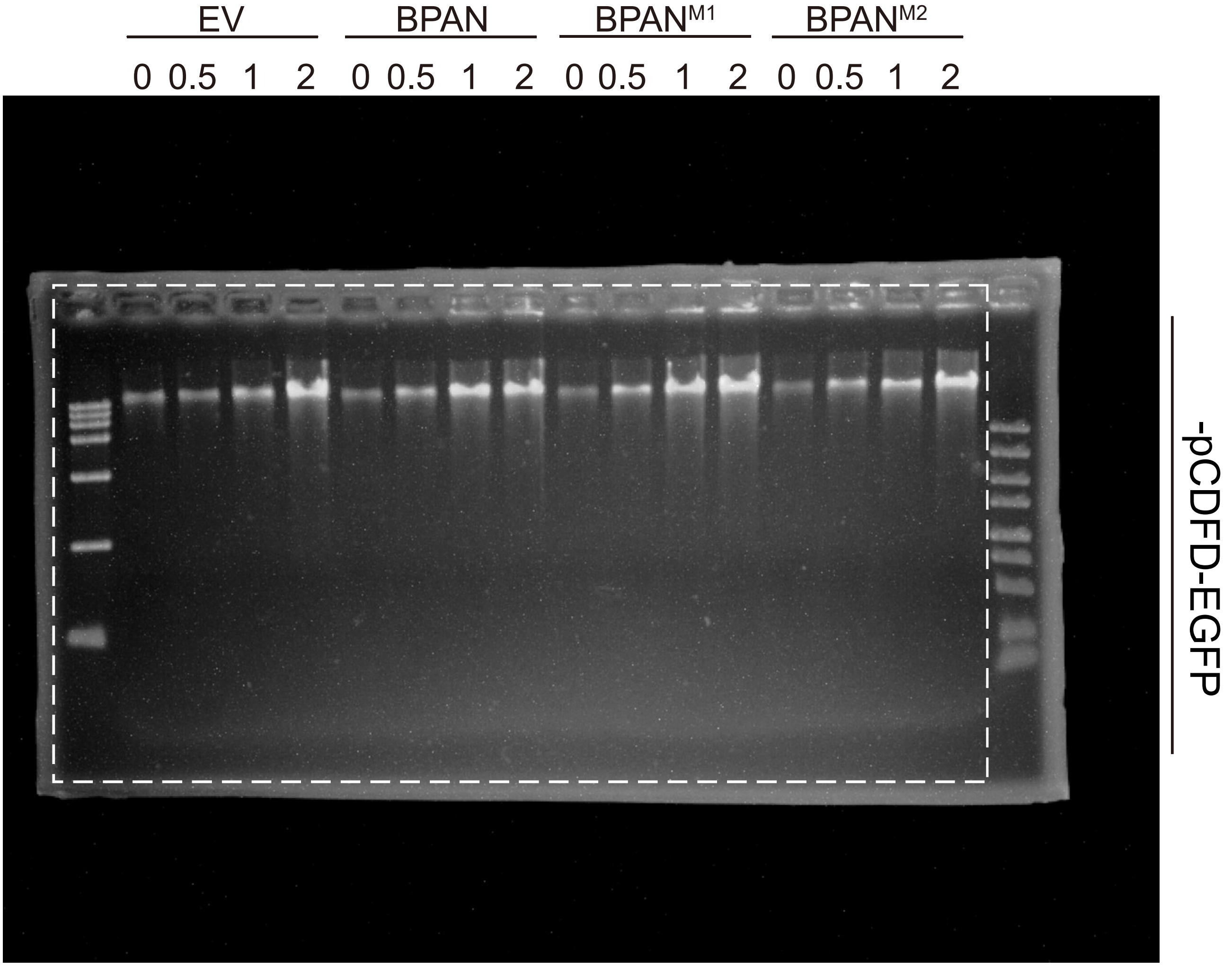

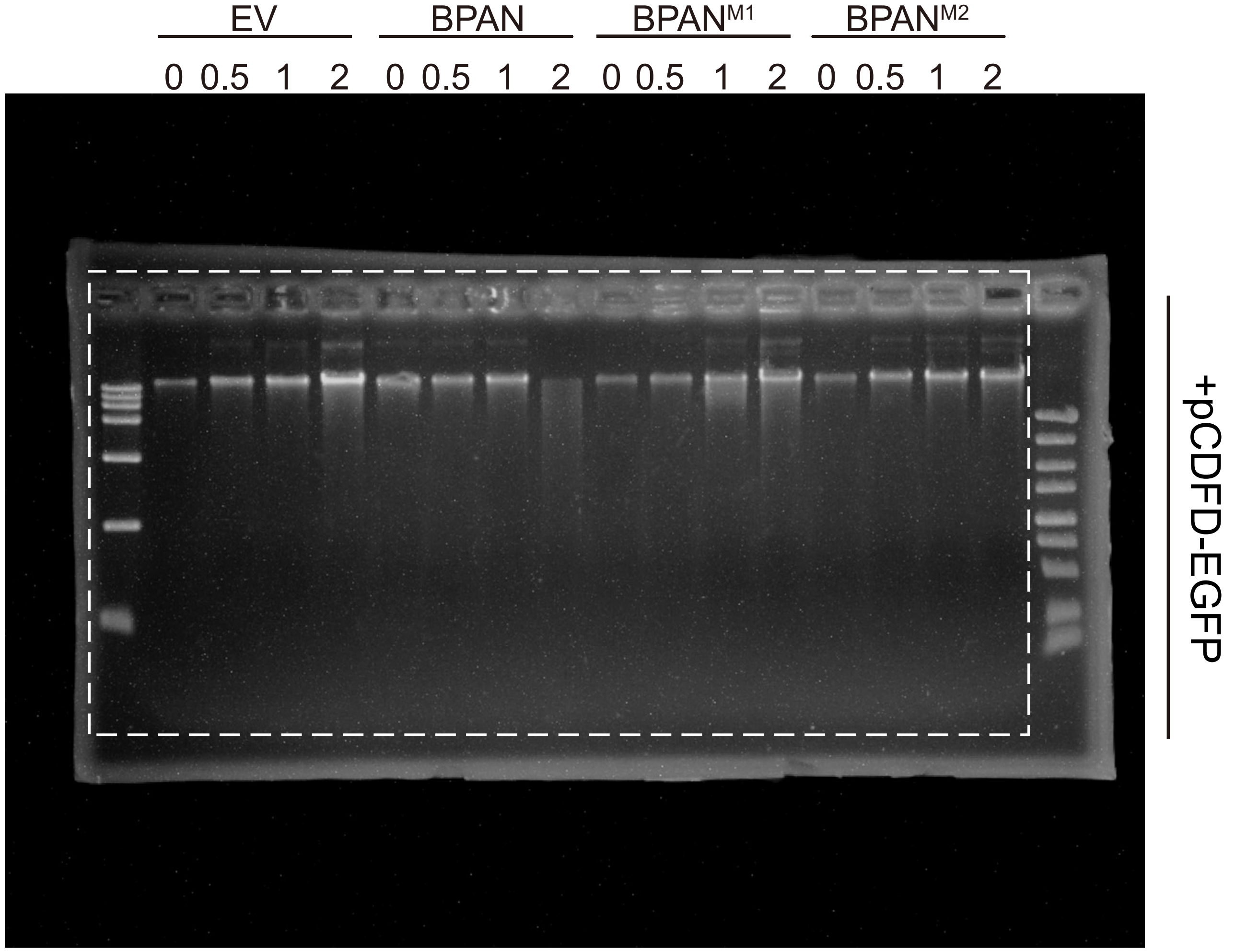

Source Data Supplementary Fig. 7b

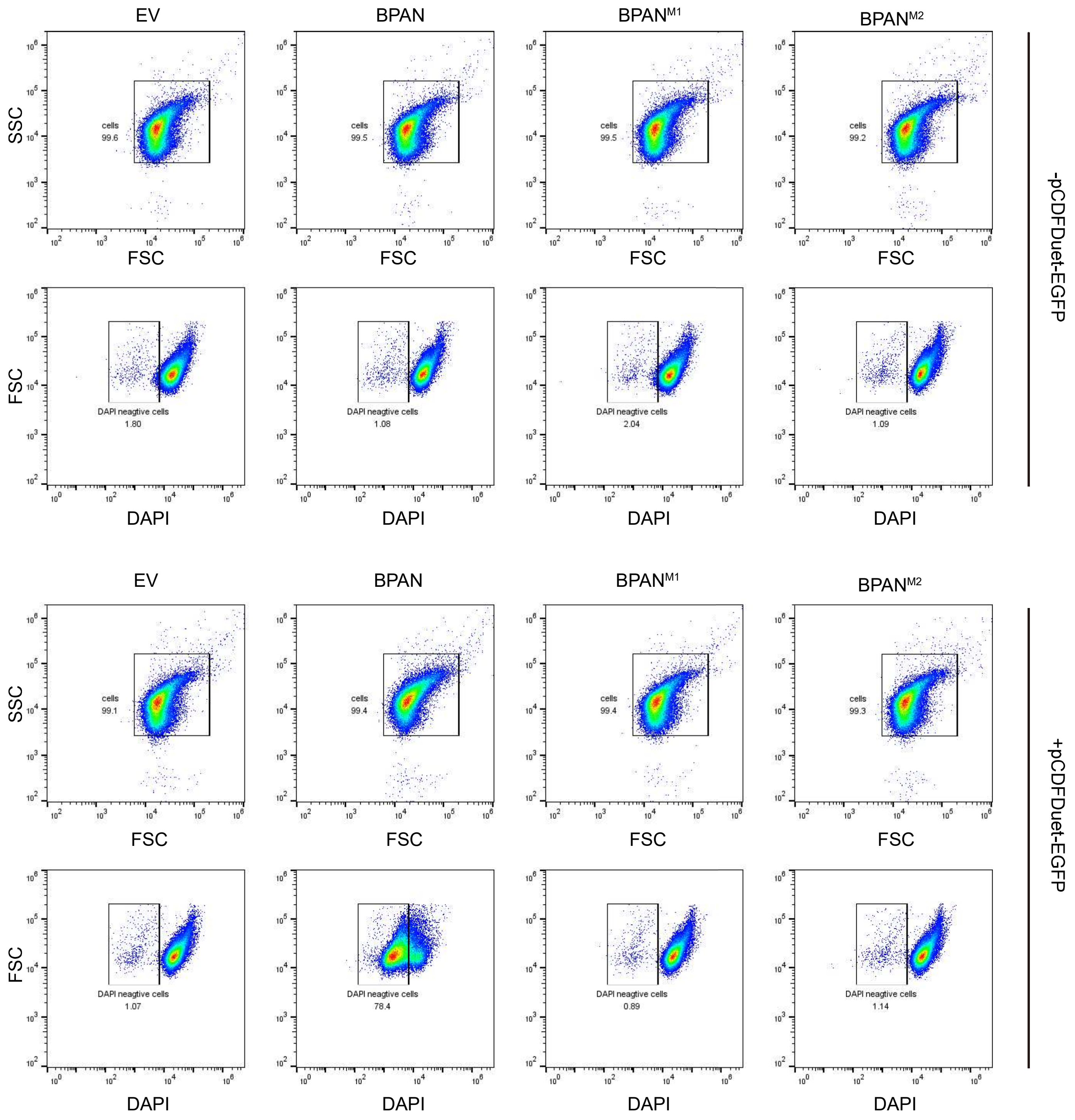

Source Data Supplementary Fig. 7d

EV

phage titre

T5

T7

λvir

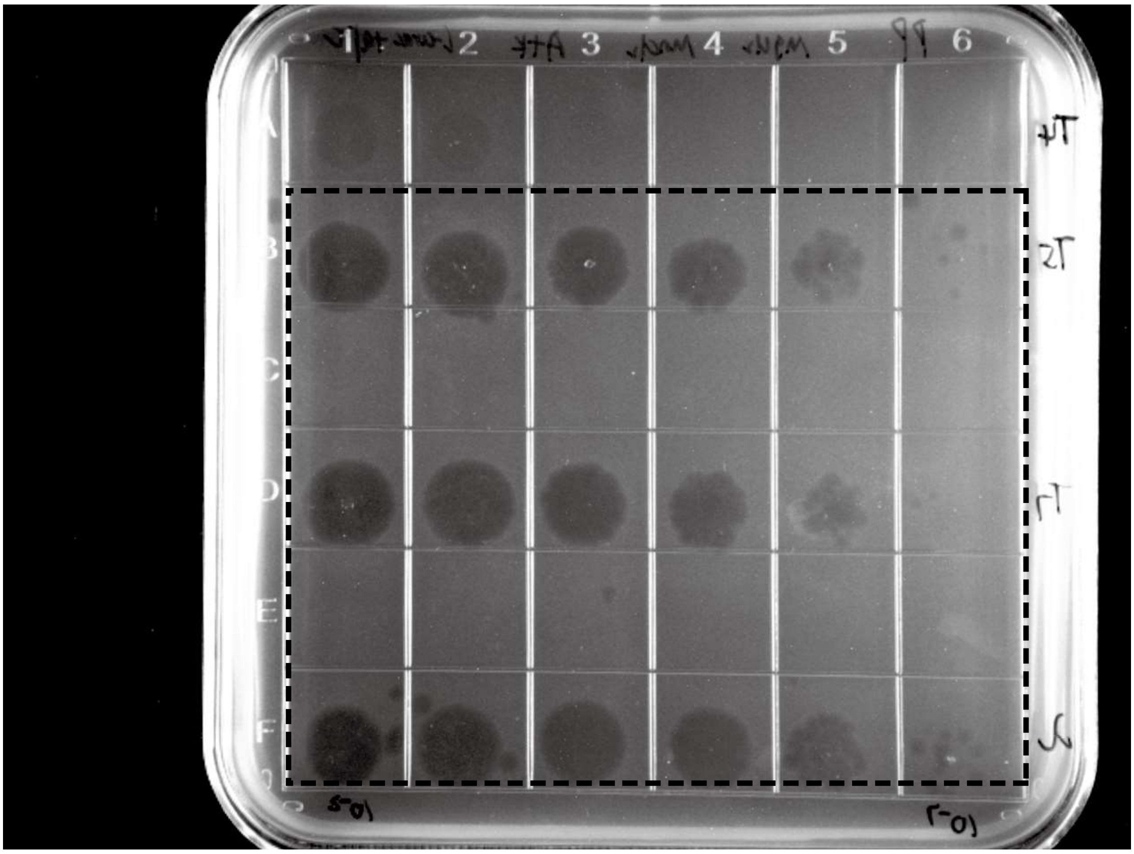

BPAN

T5

T7

λvir

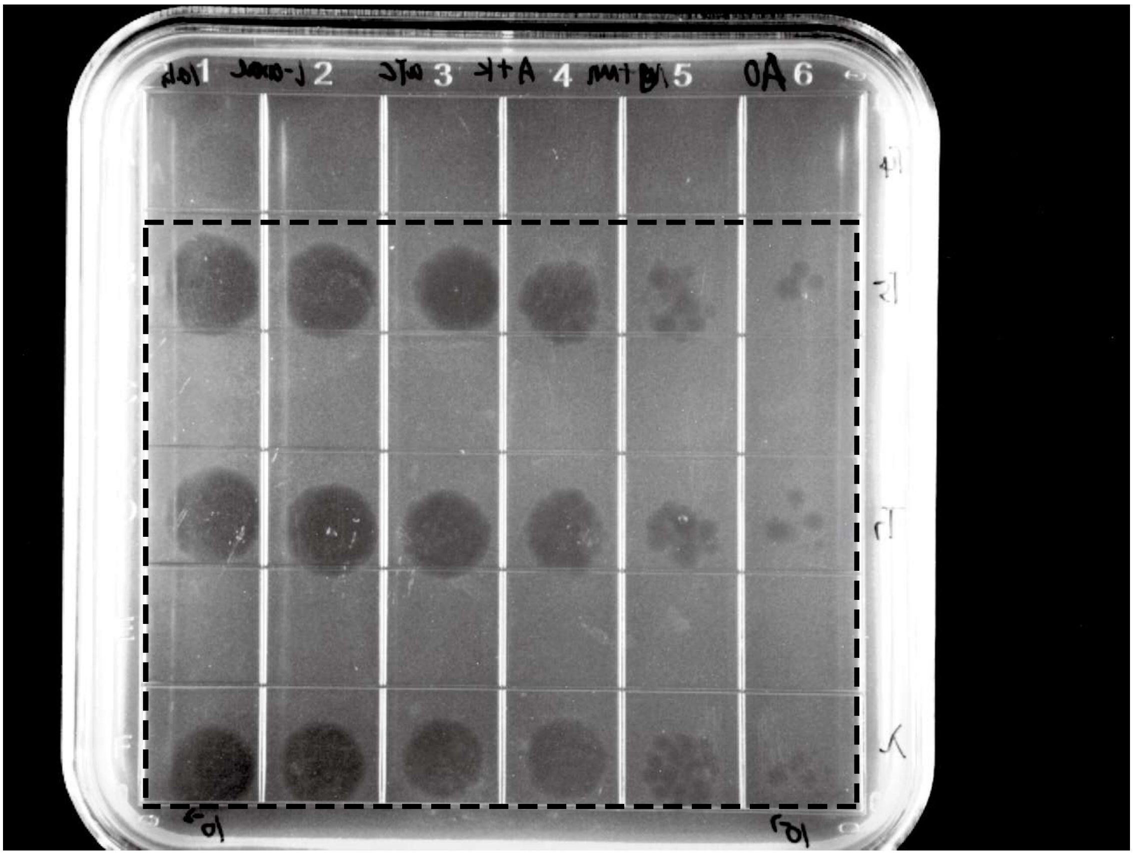

Source Data Supplementary Fig. 8a

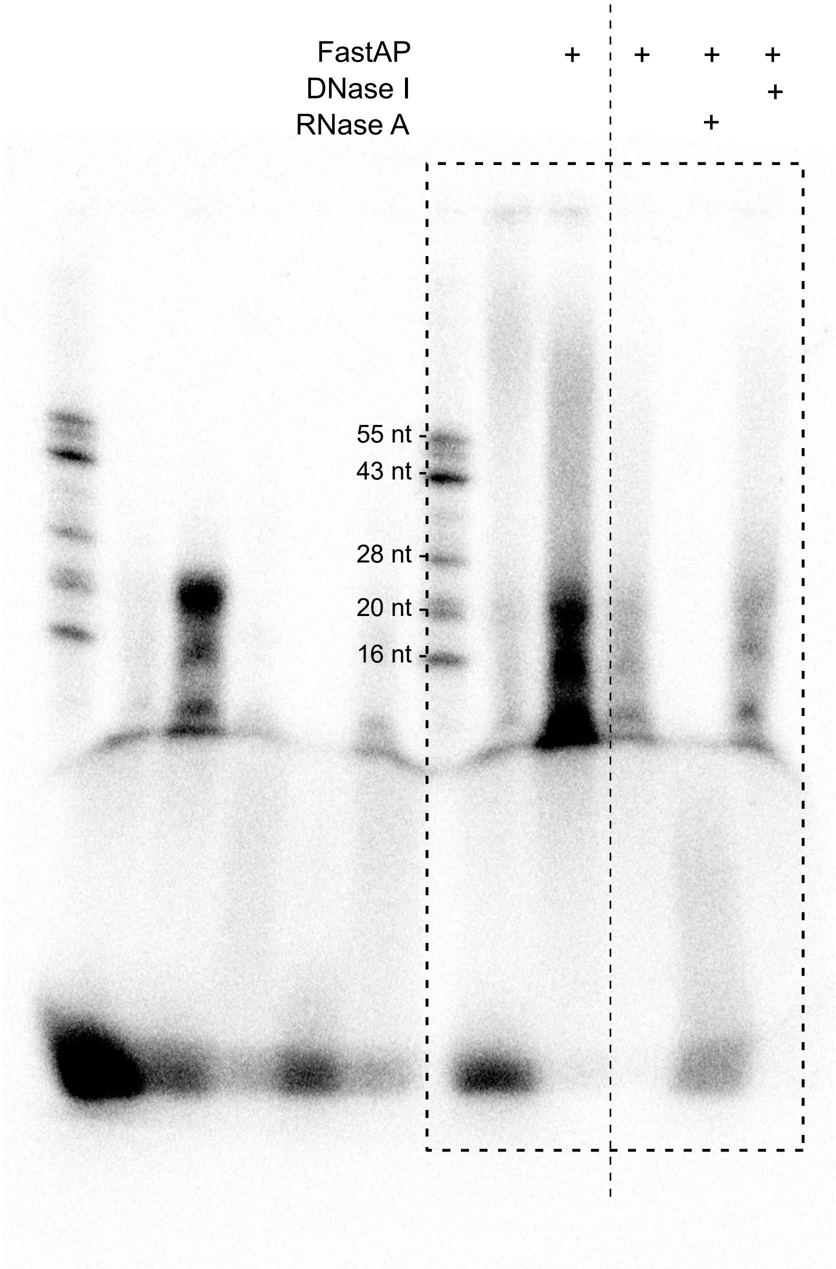

Source Data Supplementary Fig.9

a

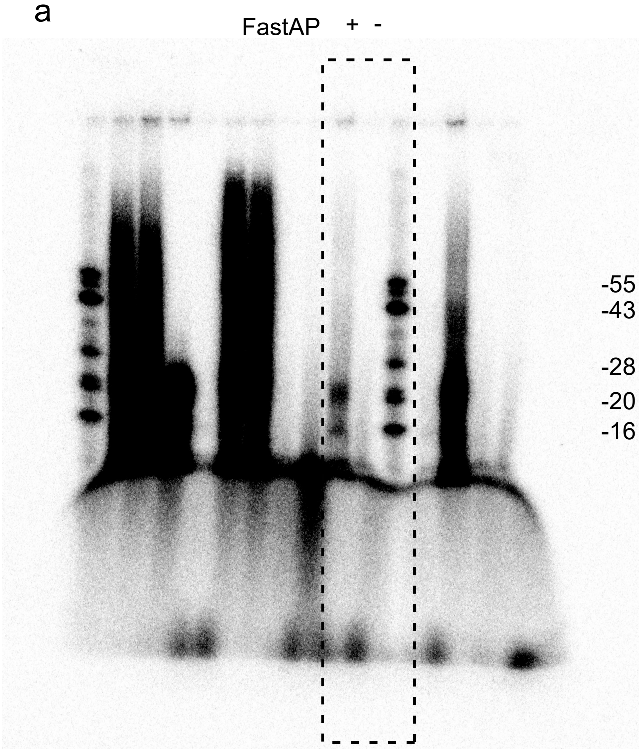

b

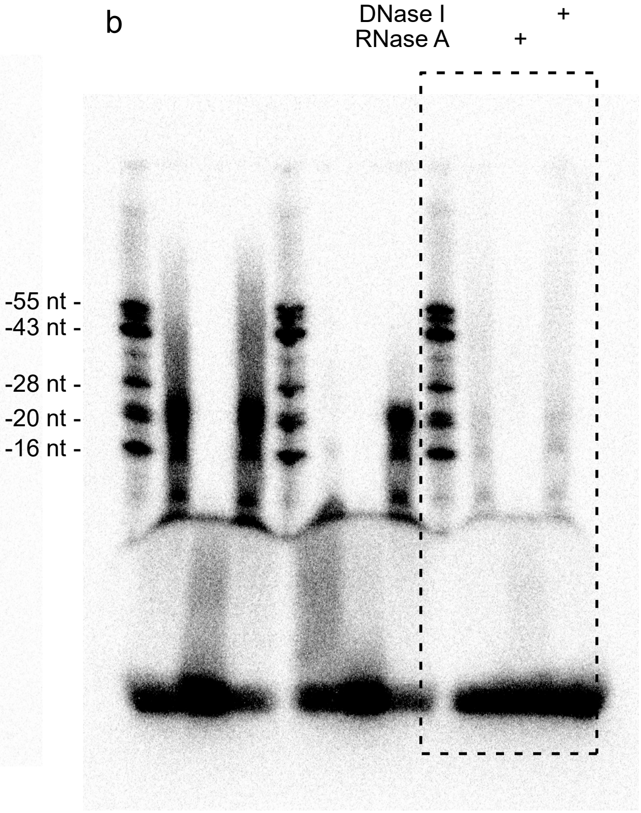

Source Data Supplementary Fig. 12

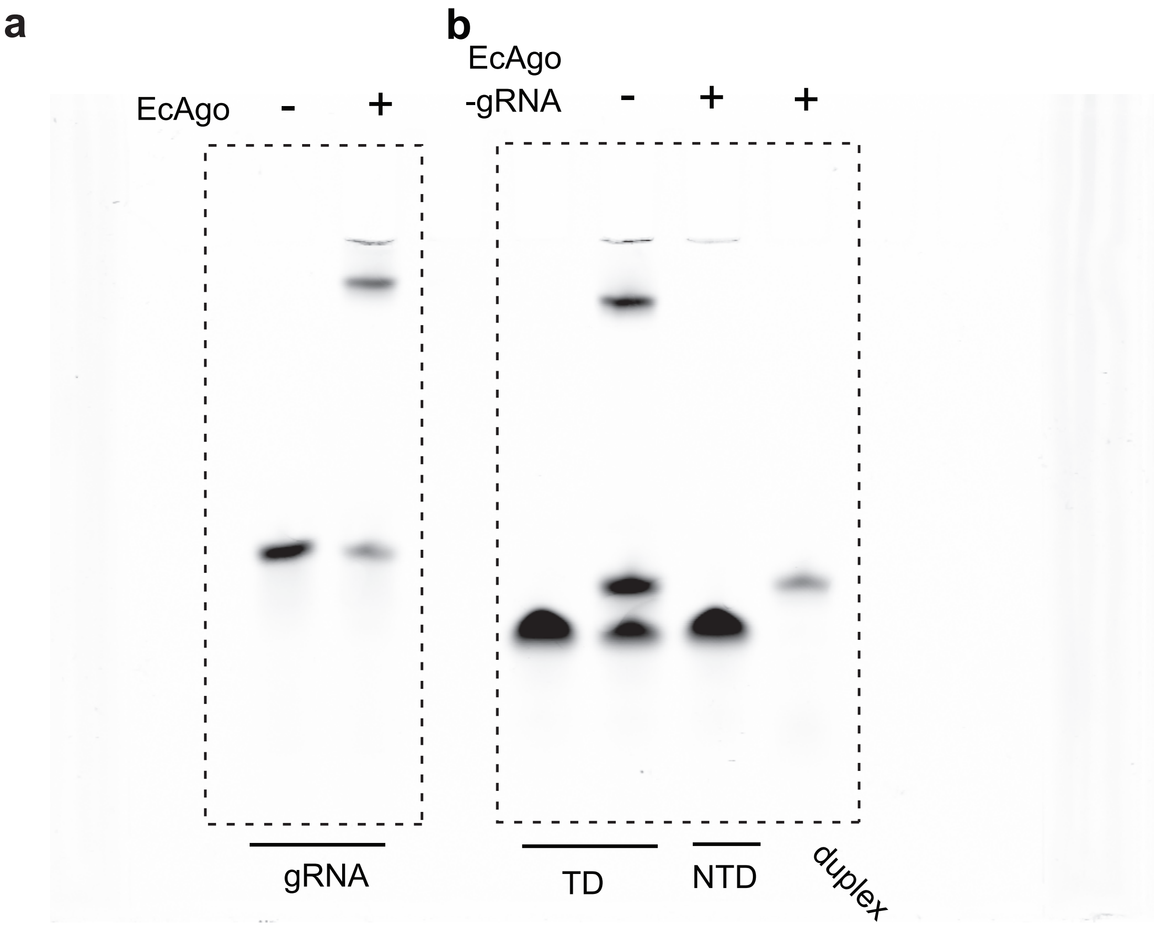

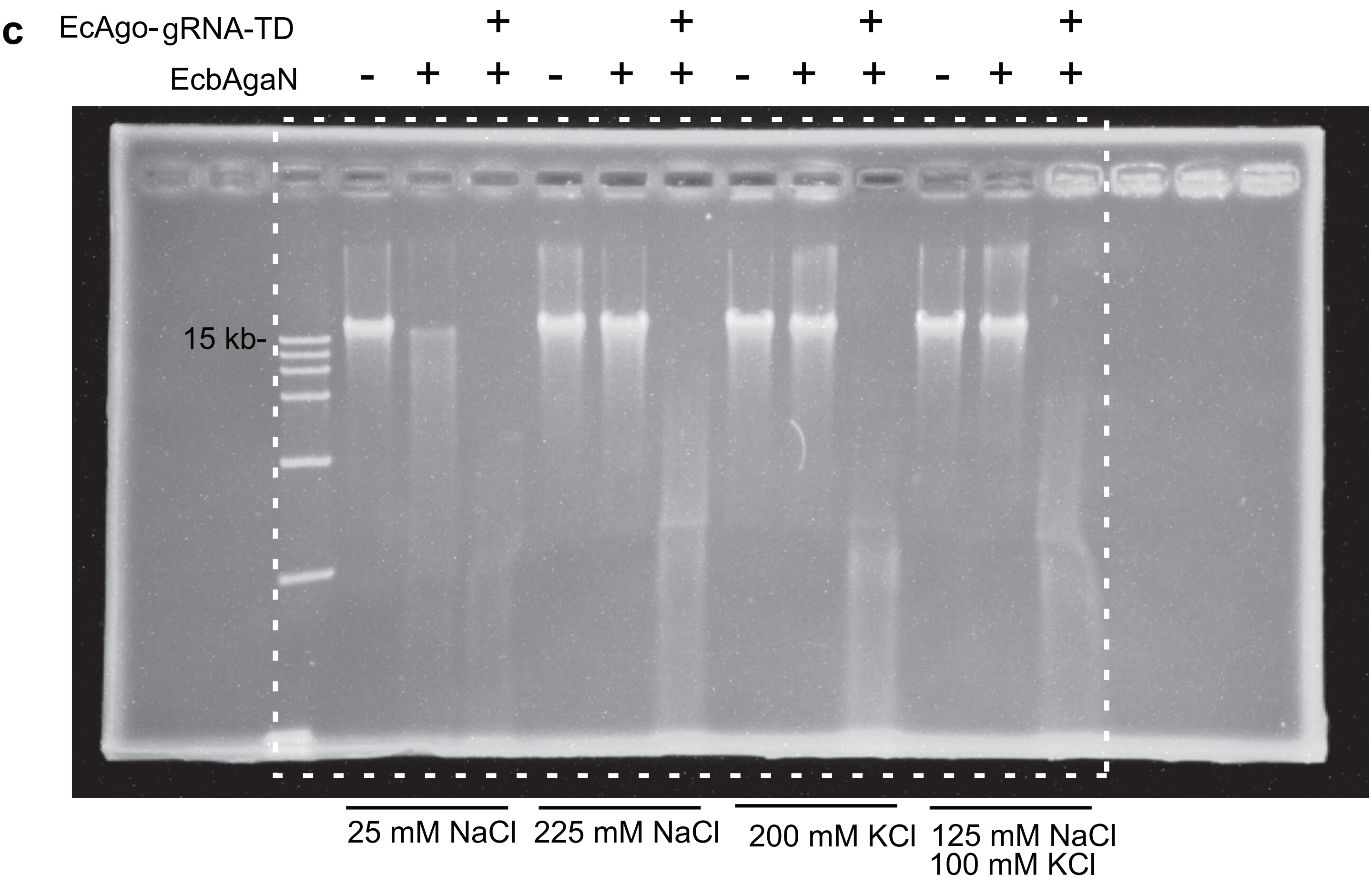

Source Data Supplementary Fig.13

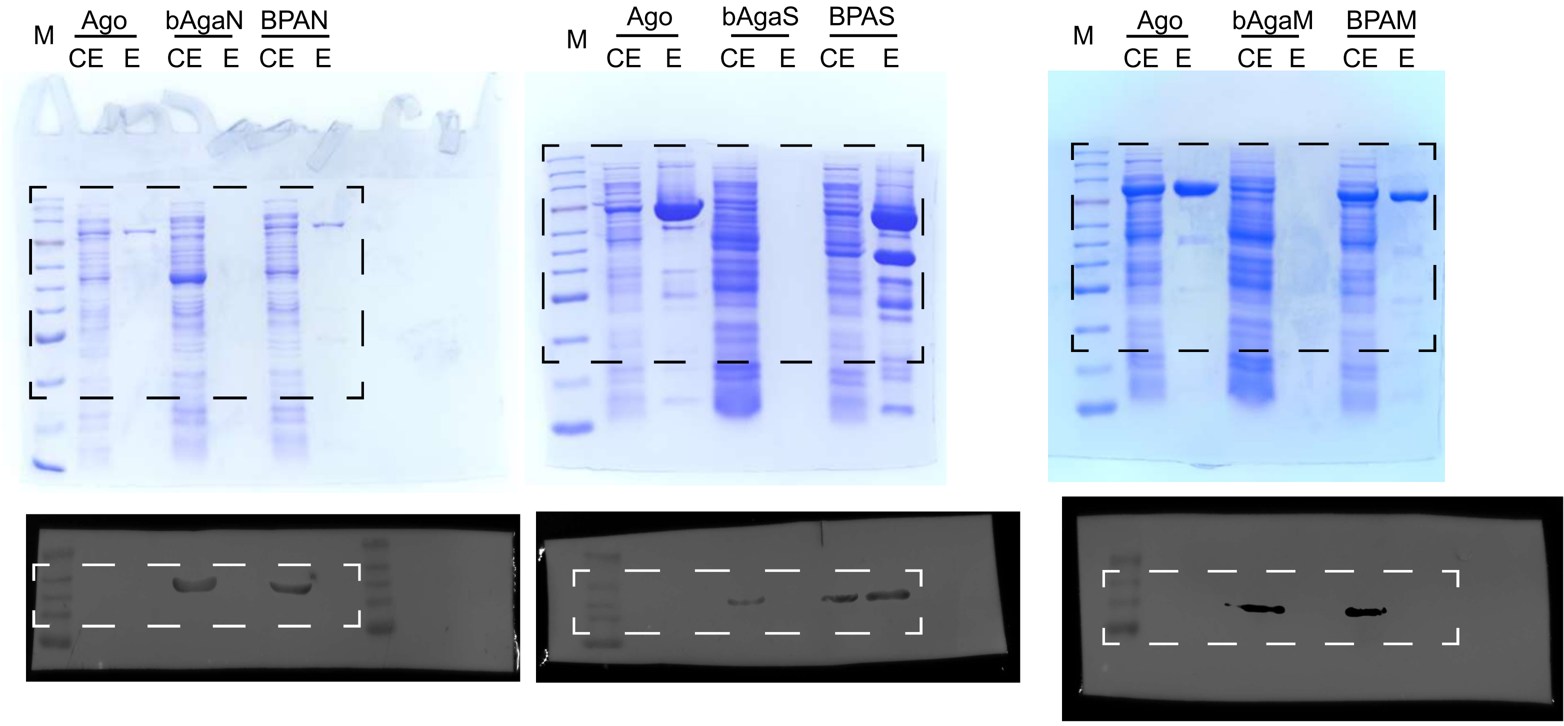

Supplement: Supplementary file 1 — Supplementary Information [file 41467_2023_42793_MOESM1_ESM.pdf]
