# Supplementary material for: Catalytically inactive long prokaryotic Argonaute systems employ distinct effectors to confer immunity via abortive infection
Source: Nat Commun. 2023 Nov 1;14:6970. doi: 10.1038/s41467-023-42793-3 (PMC10620215; doi:10.1038/s41467-023-42793-3)
Supplement: Supplementary file 3 — Description of Additional Supplementary Files [file 41467_2023_42793_MOESM3_ESM.pdf]

Description of Additional Supplementary Files

File Name: Supplementary Data 1
Description: Proteins analyzed in the study. The names, accession numbers, source strains, and sequences are listed.

File Name: Supplementary Data 2
Description: Plasmids used in the study.

File Name: Supplementary Data 3.
Description: Primers used in the study

File Name: Supplementary Data 4
Description: Oligonucleotides used in the study.

File Name: Supplementary Data 5
Description: Alignments of the sequenced RNA reads from transcriptome and EcAgo-associated small RNAs. The RNAs were from three different samples, as indicated by the plasmids carried by the cells.
