# Supplementary material for: Catalytically inactive long prokaryotic Argonaute systems employ distinct effectors to confer immunity via abortive infection
Source: Nat Commun. 2023 Nov 1;14:6970. doi: 10.1038/s41467-023-42793-3 (PMC10620215; doi:10.1038/s41467-023-42793-3)
Supplement: Supplementary file 6 — Reporting Summary [file 41467_2023_42793_MOESM6_ESM.pdf]

nature portfolio

Corresponding author(s): Wenyuan Han
Last updated by author(s): Oct 13, 2023

# Reporting Summary

Nature Portfolio wishes to improve the reproducibility of the work that we publish. This form provides structure for consistency and transparency in reporting. For further information on Nature Portfolio policies, see our Editorial Policies and the Editorial Policy Checklist.

## Statistics

For all statistical analyses, confirm that the following items are present in the figure legend, table legend, main text, or Methods section.

| n/a | Confirmed | |
|---|---|---|
| ☐ | ☒ | The exact sample size (*n*) for each experimental group/condition, given as a discrete number and unit of measurement |
| ☐ | ☒ | A statement on whether measurements were taken from distinct samples or whether the same sample was measured repeatedly |
| ☐ | ☒ | The statistical test(s) used AND whether they are one- or two-sided<br>*Only common tests should be described solely by name; describe more complex techniques in the Methods section.* |
| ☒ | ☐ | A description of all covariates tested |
| ☒ | ☐ | A description of any assumptions or corrections, such as tests of normality and adjustment for multiple comparisons |
| ☐ | ☒ | A full description of the statistical parameters including central tendency (e.g. means) or other basic estimates (e.g. regression coefficient) AND variation (e.g. standard deviation) or associated estimates of uncertainty (e.g. confidence intervals) |
| ☐ | ☒ | For null hypothesis testing, the test statistic (e.g. *F*, *t*, *r*) with confidence intervals, effect sizes, degrees of freedom and *P* value noted<br>*Give P values as exact values whenever suitable.* |
| ☒ | ☐ | For Bayesian analysis, information on the choice of priors and Markov chain Monte Carlo settings |
| ☒ | ☐ | For hierarchical and complex designs, identification of the appropriate level for tests and full reporting of outcomes |
| ☐ | ☒ | Estimates of effect sizes (e.g. Cohen's *d*, Pearson's *r*), indicating how they were calculated |

*Our web collection on statistics for biologists contains articles on many of the points above.*

## Software and code

Policy information about availability of computer code

| | |
|---|---|
| Data collection | Flow cytometry data were collected with FlowJo v.10.8.1. SEC-MALS data were collected with ASTRA 7 software. |
| Data analysis | FlowJo v10.8.1; ASTRA 7; UNICORN 7; Excel 2007; Origin 2018; AlphaFold2 implemented in COSMIC2 platform; Clustal W; ESPript 3.0; DeepTMHMM; PyMOL; Graphpad Prism 8.0.1; fastp 0.23.1; hisat2 2.1.0; bowtie2 2.2.5; samtools 1.16.1; seqkit 2.3.0; iTOL 6.5.8; featureCounts 2.0.1; MAFFT 7.508; FastTree 2.1.11; ggplot2(R package) 3.3.6; ape(R package) 5.6-2; ggseqlogo(R package) 0.1; NCBI blast |

For manuscripts utilizing custom algorithms or software that are central to the research but not yet described in published literature, software must be made available to editors and reviewers. We strongly encourage code deposition in a community repository (e.g. GitHub). See the Nature Portfolio guidelines for submitting code & software for further information.

## Data

Policy information about availability of data

All manuscripts must include a data availability statement. This statement should provide the following information, where applicable:

- Accession codes, unique identifiers, or web links for publicly available datasets
- A description of any restrictions on data availability
- For clinical datasets or third party data, please ensure that the statement adheres to our policy

The data are available in the manuscript, supplementary materials and raw dataset. In addition, small and total RNA sequencing data are available on the NCBI Sequence Read Archive under BioProject ID PRJNA1003211 (https://www.ncbi.nlm.nih.gov/bioproject/PRJNA1003211).

## Research involving human participants, their data, or biological material

Policy information about studies with human participants or human data. See also policy information about sex, gender (identity/presentation), and sexual orientation and race, ethnicity and racism.

| | |
|---|---|
| Reporting on sex and gender | N/A |
| Reporting on race, ethnicity, or other socially relevant groupings | N/A |
| Population characteristics | N/A |
| Recruitment | N/A |
| Ethics oversight | N/A |

Note that full information on the approval of the study protocol must also be provided in the manuscript.

# Field-specific reporting

Please select the one below that is the best fit for your research. If you are not sure, read the appropriate sections before making your selection.

☒ Life sciences ☐ Behavioural & social sciences ☐ Ecological, evolutionary & environmental sciences

For a reference copy of the document with all sections, see nature.com/documents/nr-reporting-summary-flat.pdf

# Life sciences study design

All studies must disclose on these points even when the disclosure is negative.

| | |
|---|---|
| Sample size | Experiments were performed in at least 3 independent replicates (n=3 or 5 as indicated in the figure legends). The sample size is determined following the standard experimental designs in this field, without pre-calculation of the sample size. The regular sample size of 3 (or 5) was selected, which is sufficient to avoid random variations. |
| Data exclusions | No data was excluded. |
| Replication | Reproducibility was ensured by at least 3 independent replicates. |
| Randomization | Bacterial colonies were randomly selected for biological replicates. |
| Blinding | Blinding was not relevant to this study, since no manual counting or scoring was performed to obtain data. |

# Reporting for specific materials, systems and methods

We require information from authors about some types of materials, experimental systems and methods used in many studies. Here, indicate whether each material, system or method listed is relevant to your study. If you are not sure if a list item applies to your research, read the appropriate section before selecting a response.

## Materials & experimental systems

| n/a | Involved in the study | |
|---|---|---|
| ☐ | ☒ | Antibodies |
| ☒ | ☐ | Eukaryotic cell lines |
| ☒ | ☐ | Palaeontology and archaeology |
| ☒ | ☐ | Animals and other organisms |
| ☒ | ☐ | Clinical data |
| ☒ | ☐ | Dual use research of concern |
| ☒ | ☐ | Plants |

## Methods

| n/a | Involved in the study | |
|---|---|---|
| ☒ | ☐ | ChIP-seq |
| ☐ | ☒ | Flow cytometry |
| ☒ | ☐ | MRI-based neuroimaging |

## Antibodies

| | |
|---|---|
| Antibodies used | Anti-His Tag Rabbit Polyclonal Antibody (ABT2051, Abbkine, Wuhan, China); Anti-HA Tag Rabbit Polyclonal Antibody (ABT2041, Abbkine, Wuhan, China); HRP, Goat Anti-Rabbit IgG (A21020, Abbkine, Wuhan, China) |

Validation

https://www.abbkine.com/product/anti-his-tag-rabbit-polyclonal-antibody-abt2051/;
https://www.abbkine.com/product/anti-ha-tag-rabbit-polyclonal-antibody-abt2041/;
https://www.abbkine.com/product/hrp-goat-anti-rabbit-igg-a21020/

## Plants

| | |
|---|---|
| Seed stocks | N/A |
| Novel plant genotypes | N/A |
| Authentication | N/A |

## Flow Cytometry

### Plots

Confirm that:

☒ The axis labels state the marker and fluorochrome used (e.g. CD4-FITC).

☒ The axis scales are clearly visible. Include numbers along axes only for bottom left plot of group (a 'group' is an analysis of identical markers).

☒ All plots are contour plots with outliers or pseudocolor plots.

☒ A numerical value for number of cells or percentage (with statistics) is provided.

### Methodology

| | |
|---|---|
| Sample preparation | For DAPI-staining, the cells were fixed with 70% (v/v) ethanol at 4 oC overnight and washed with 1 ml lx PBS buffer for one time. |
| Instrument | cytoflex-LX flow cytometer |
| Software | FlowJo v.10.8.1 |
| Cell population abundance | At least 20,000 cells for each sample. |
| Gating strategy | Cell population is identified in the FSC-SSC plots by removal of the debris at the bottom of the plots, which also appear in the plots of the cell-free control samples.<br>The cells with the DAPI singal <5k are identified as DAPI-negative cells that have experienced genomic DNA degradation. |

☒ Tick this box to confirm that a figure exemplifying the gating strategy is provided in the Supplementary Information.
