# Supplementary figures and images for: Catalytically inactive long prokaryotic Argonaute systems employ distinct effectors to confer immunity via abortive infection
Source: Nat Commun. 2023 Nov 1;14:6970. doi: 10.1038/s41467-023-42793-3 (PMC10620215; doi:10.1038/s41467-023-42793-3)

Source Data Fig.2 b

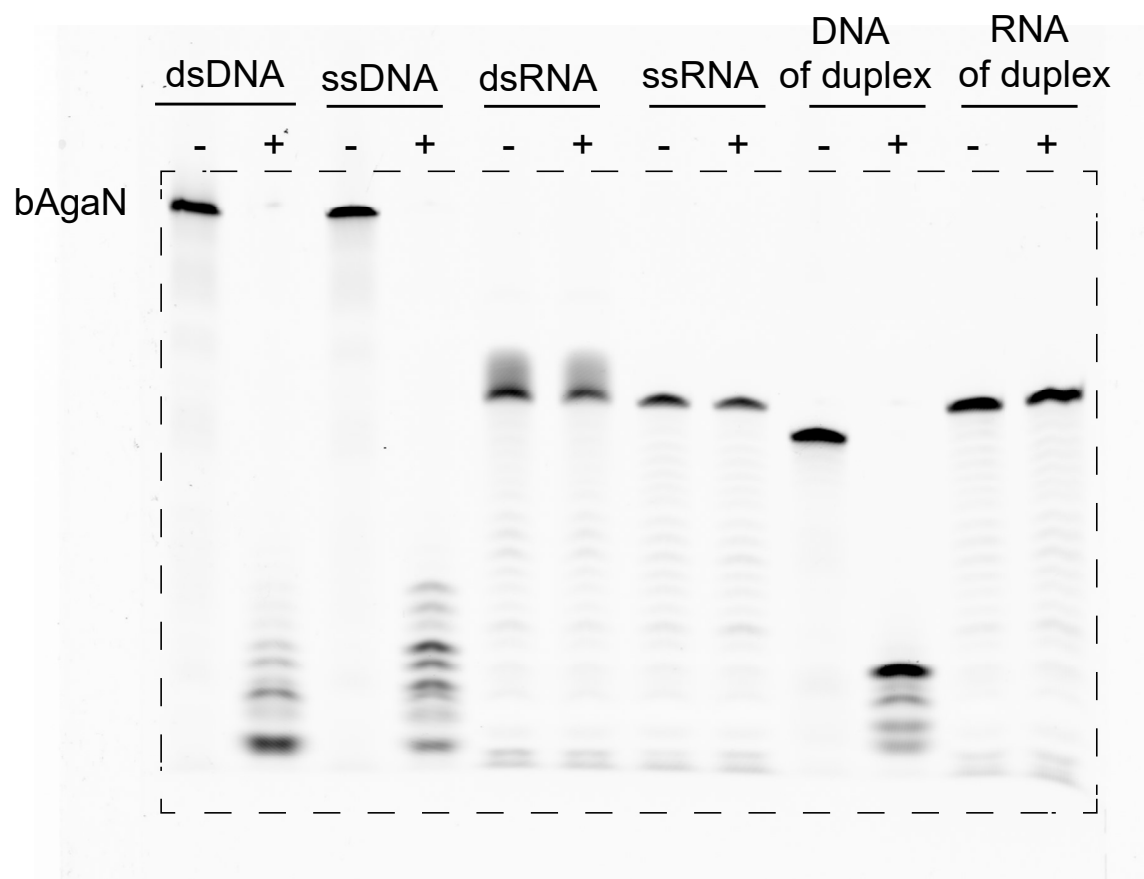

Source Data Fig.2 c

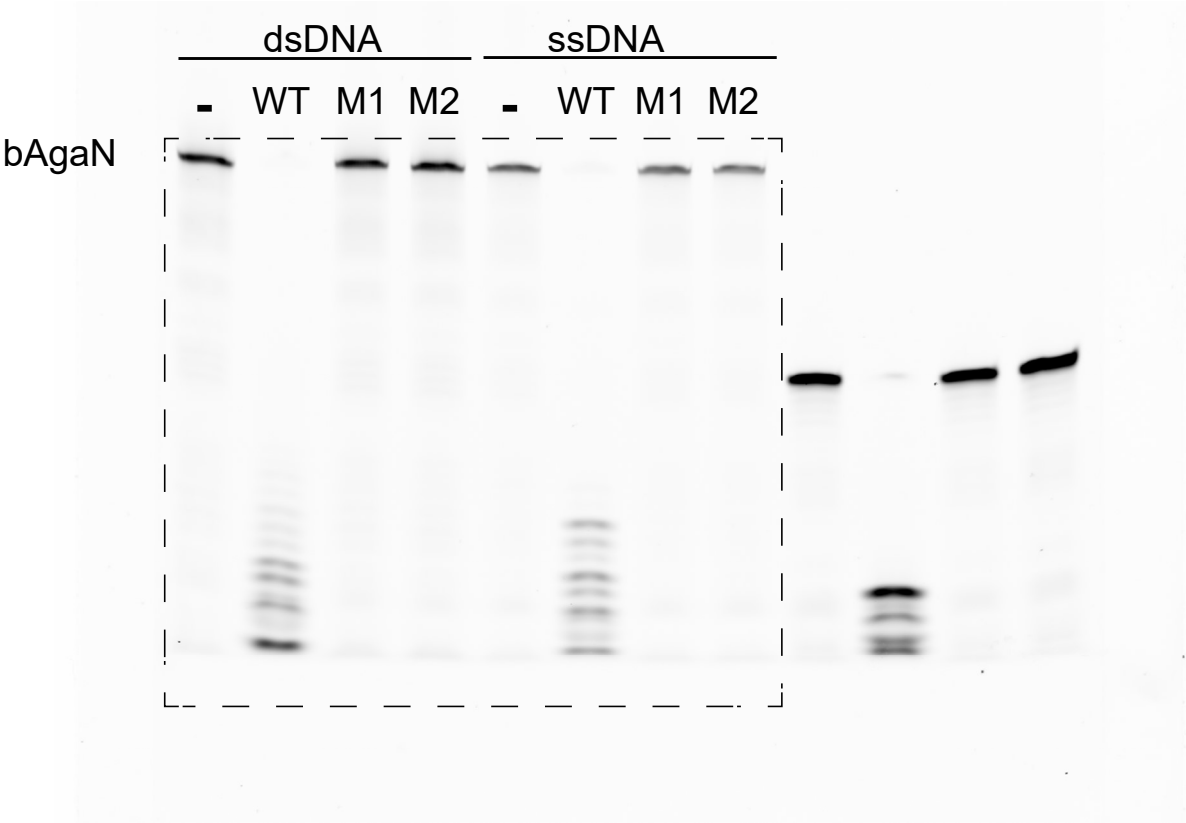

Source Data Fig. 2d

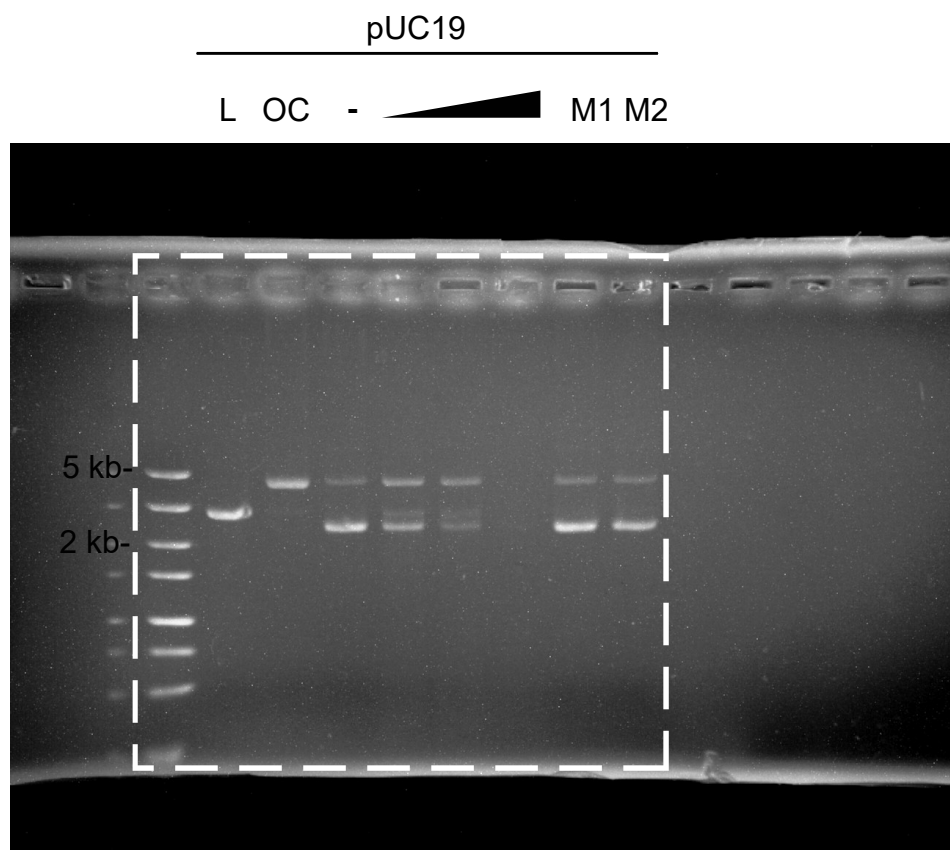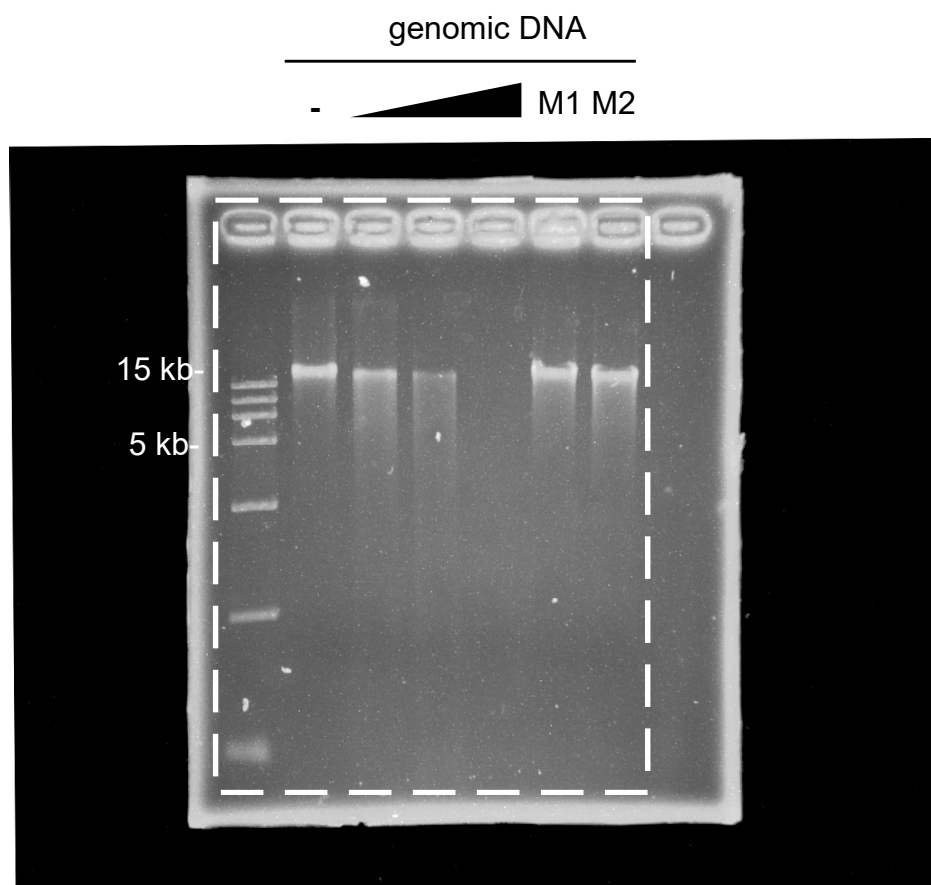

Supplement: Supplementary file 7 — Source Data [file 41467_2023_42793_MOESM7_ESM.zip › source data-Fig.2.pdf]

Source Data Fig. 3b

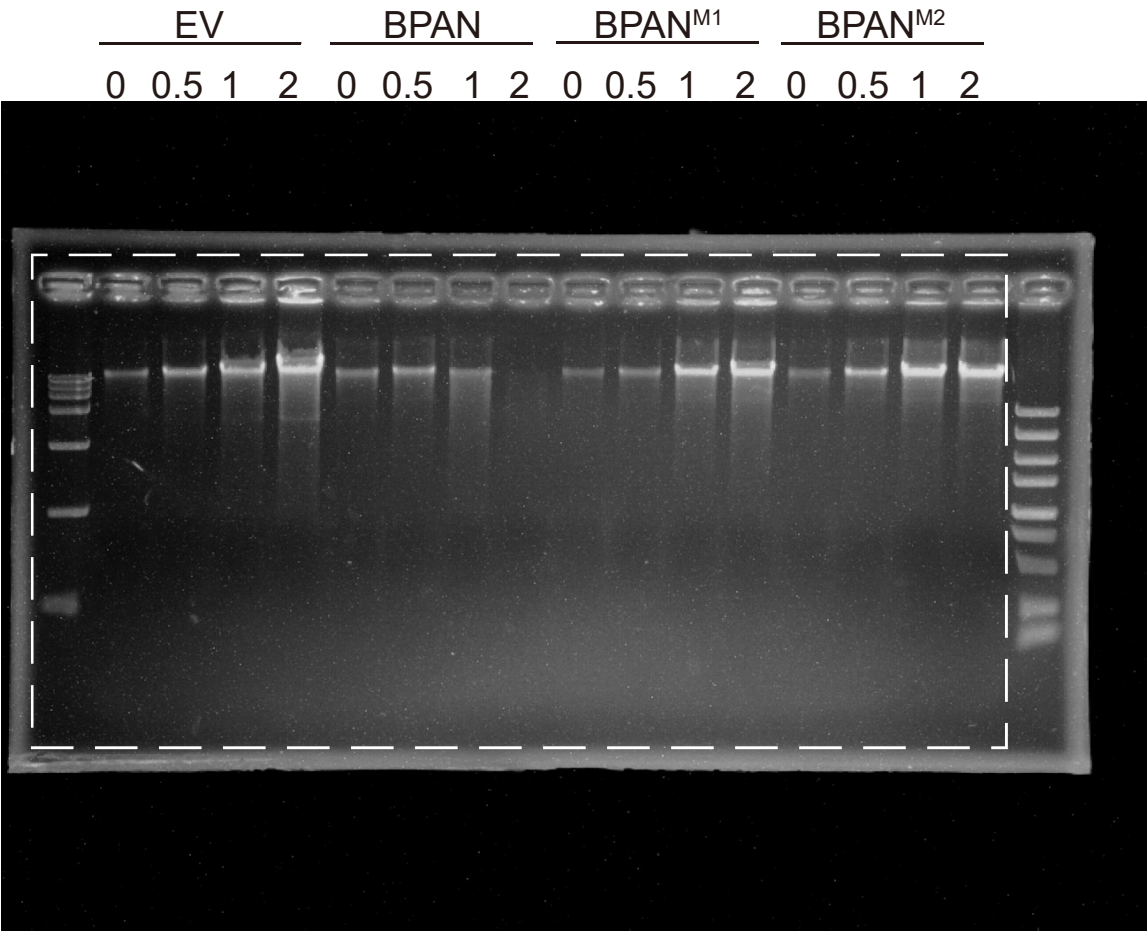

Source Data Fig. 3c

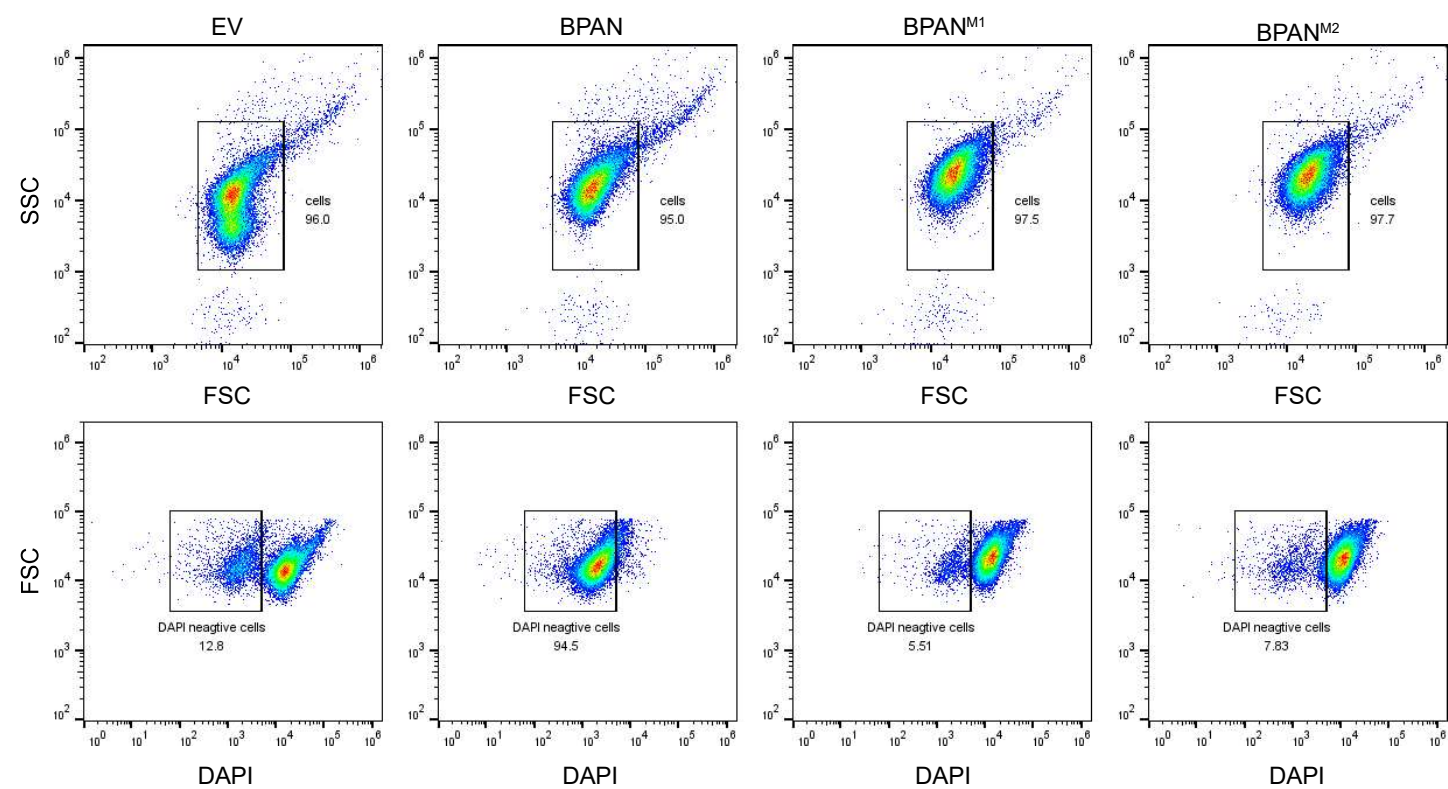

Supplement: Supplementary file 7 — Source Data [file 41467_2023_42793_MOESM7_ESM.zip › source data-Fig.3.pdf]

Source Data Fig. 4a

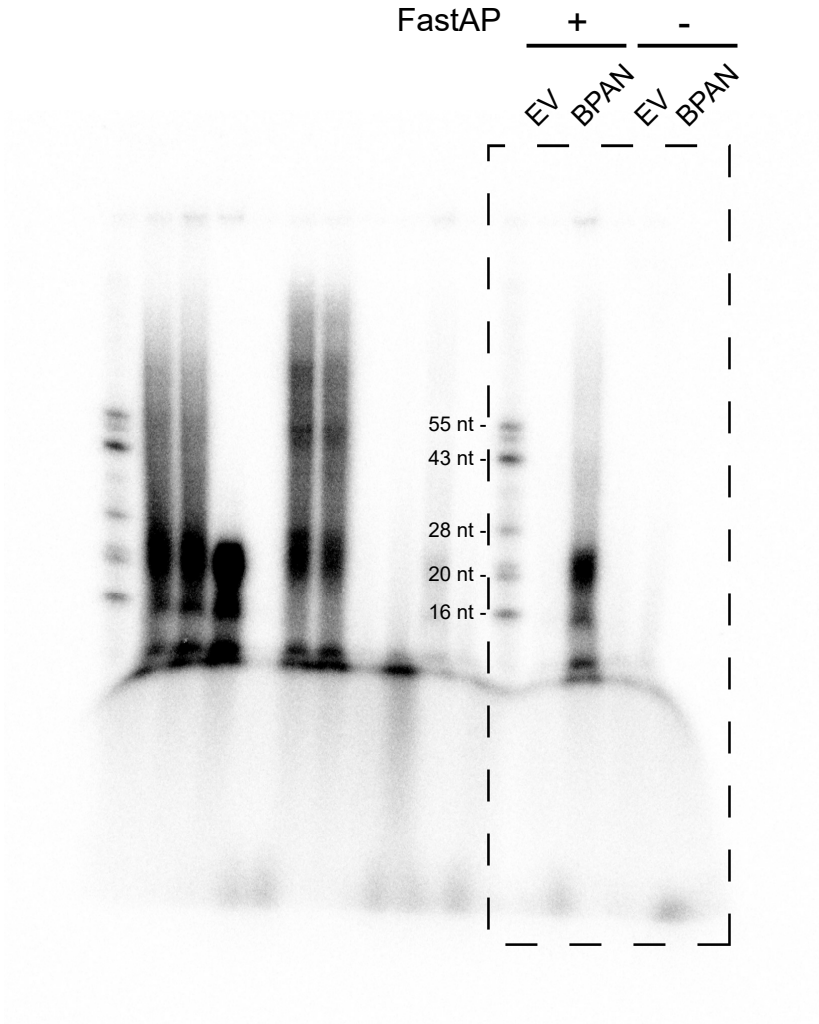

## Source Data Fig. 4b

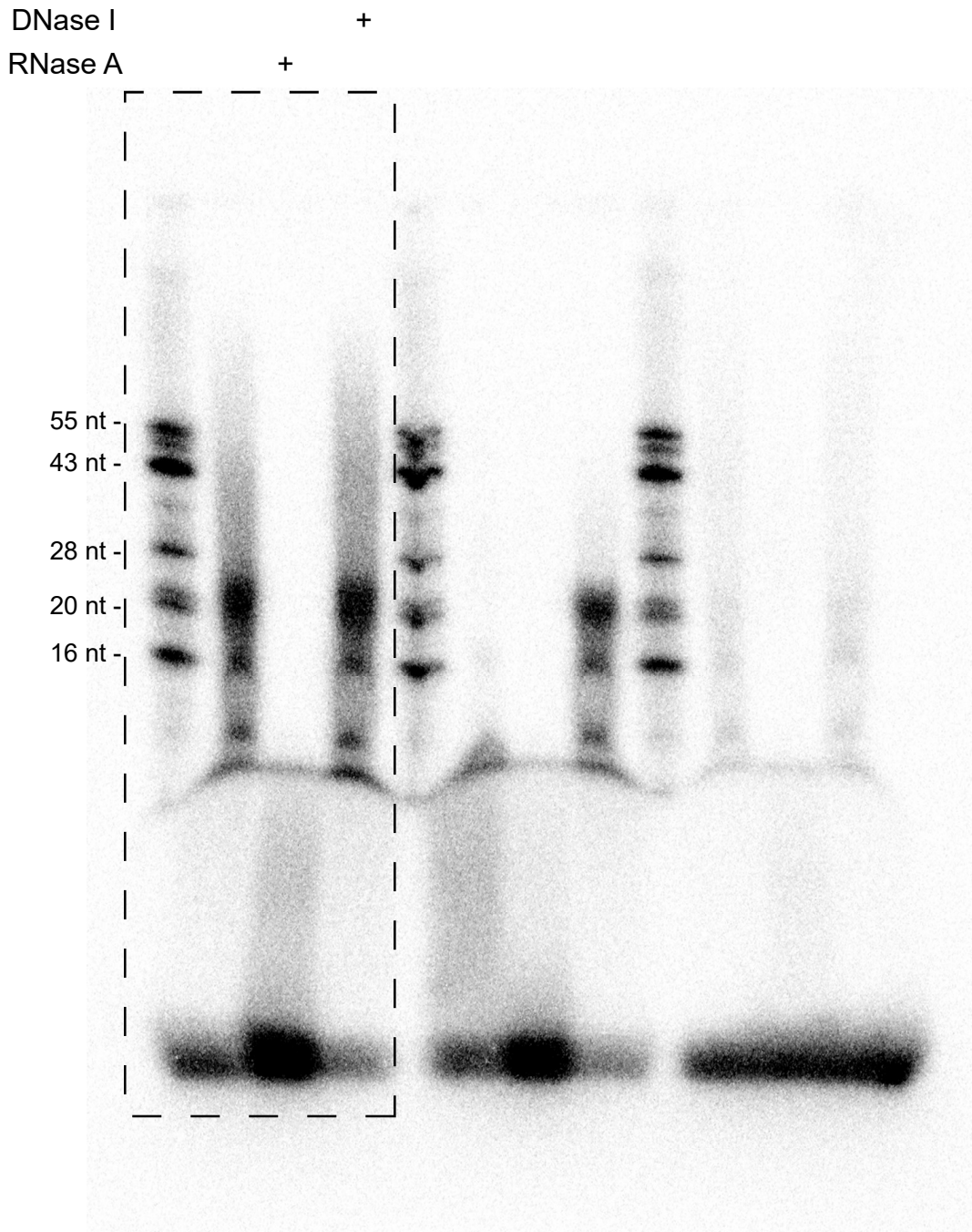

Supplement: Supplementary file 7 — Source Data [file 41467_2023_42793_MOESM7_ESM.zip › source data-Fig.4.pdf]

Source Data Fig. 5a

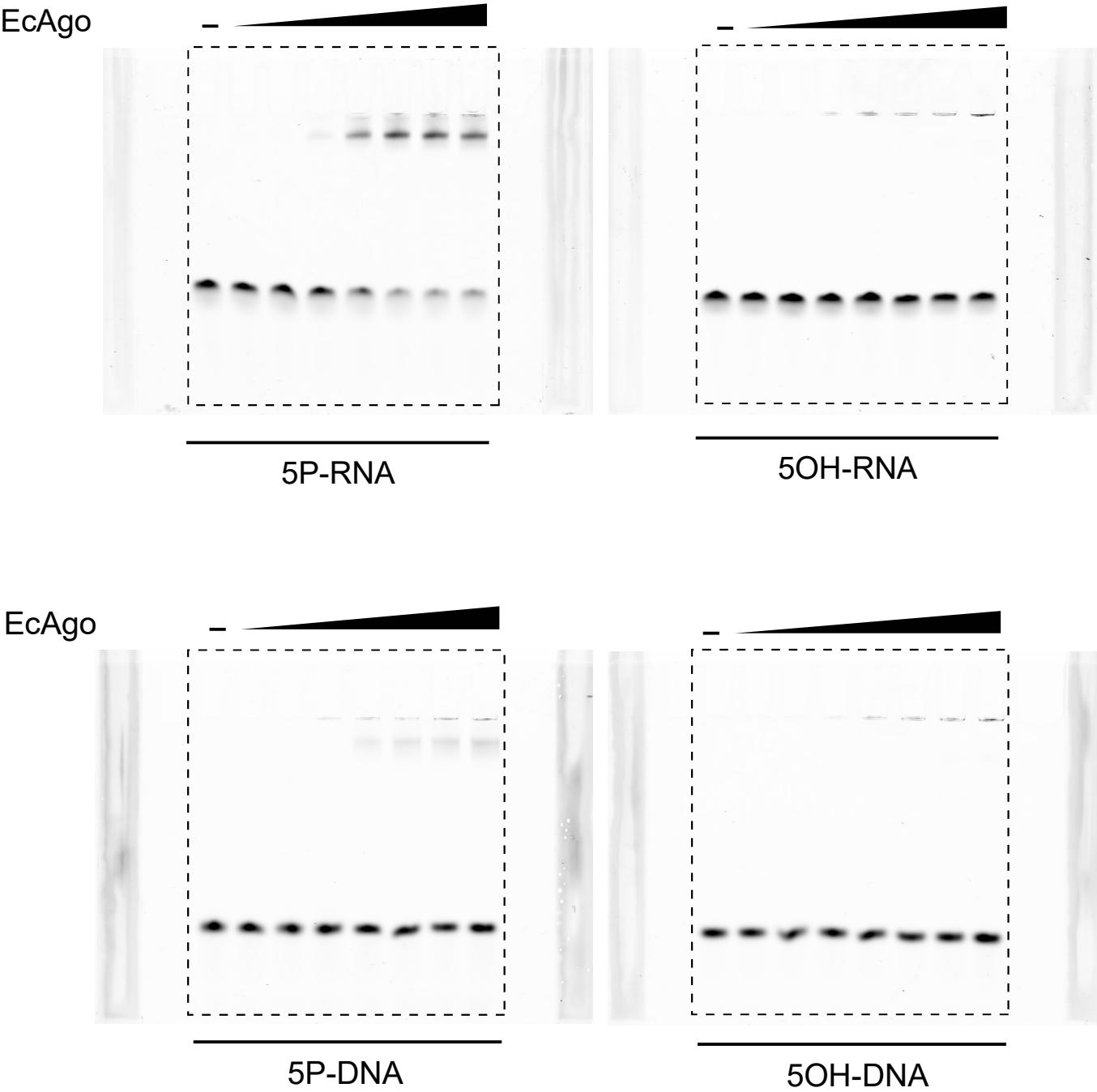

Source Data Fig. 5b

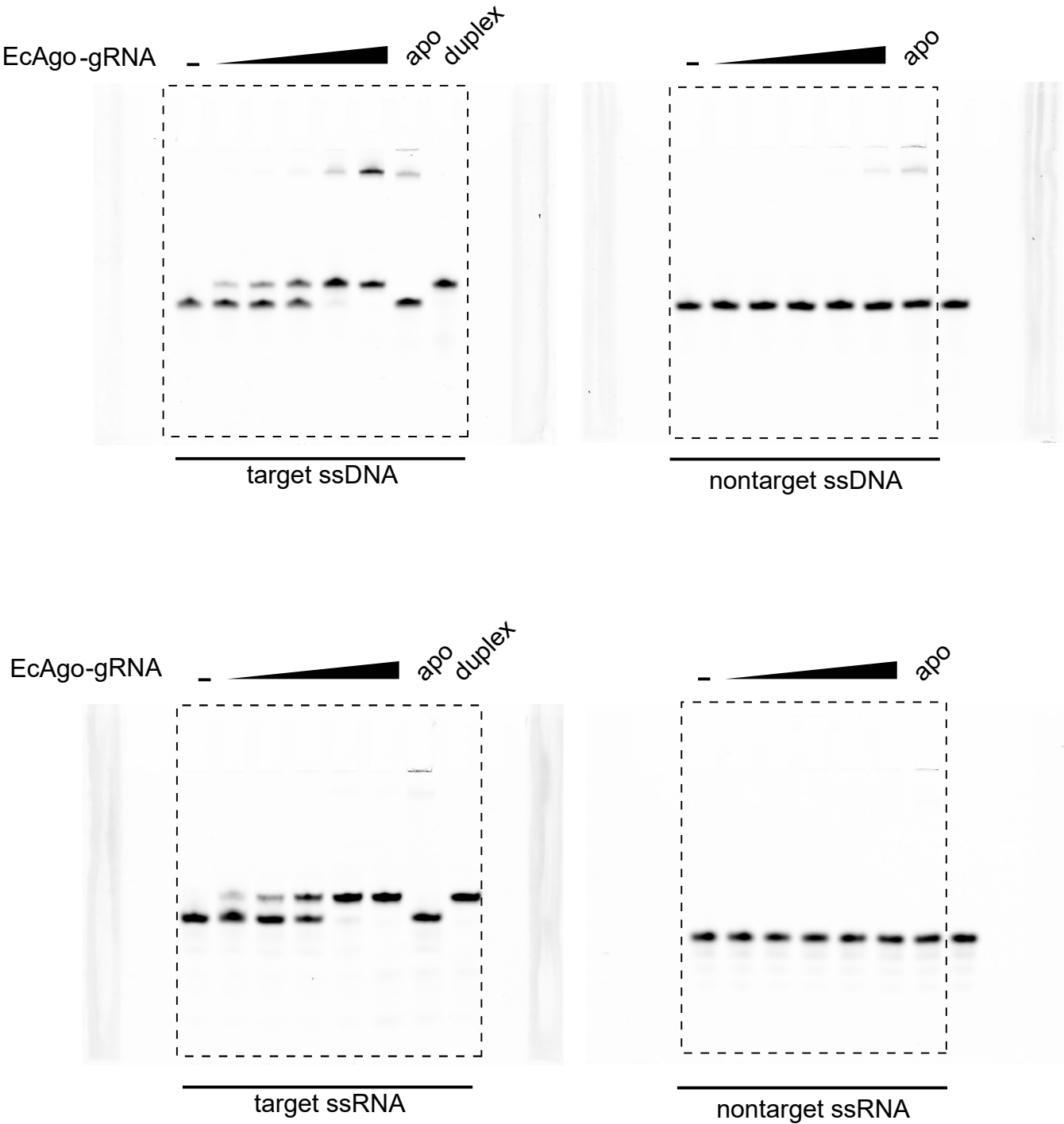

Source Data Fig. 5c

|       |   |   |   |   |   |    |     |    |     |    |    |    |
|-------|---|---|---|---|---|----|-----|----|-----|----|----|----|
| gRNA  | - | - | - | - | + | +  | +   | +  | +   | +  | +  | +  |
| EcAgo | - | + | - | + | + | +  | +   | +  | +   | +  | +  | +  |
| bAgaN | - | - | + | + | + | +  | +   | +  | +   | -  | M1 | M2 |
|       |   |   |   |   |   | TD | NTD | TR | NTR | TD | TD | TD |

15 kb-

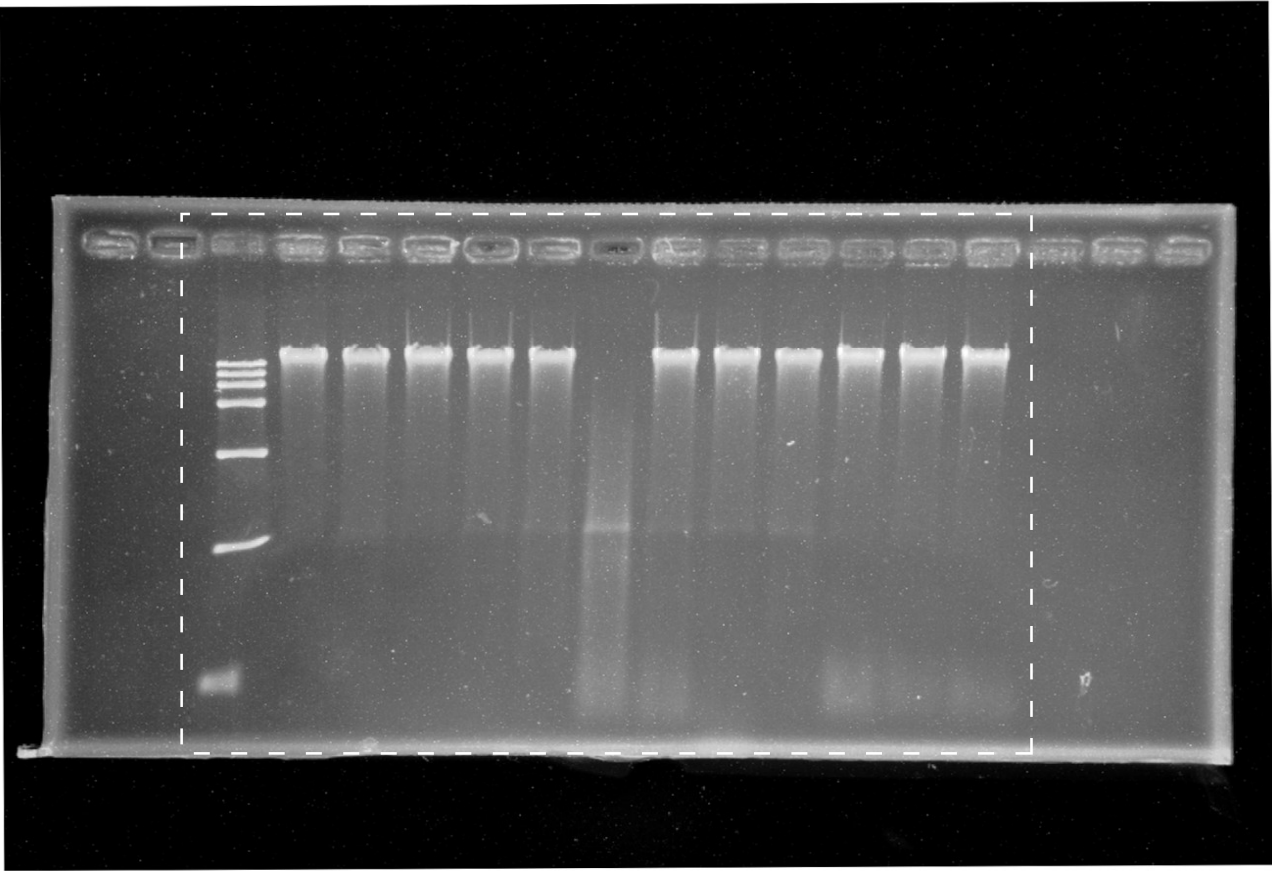

Supplement: Supplementary file 7 — Source Data [file 41467_2023_42793_MOESM7_ESM.zip › source data-Fig.5.pdf]
